# Compositional and Nutritional Analyses of Samh (*Mesembryanthemum cryptanthum*) Flour: Integrated Phenolic, Metabolomic, Fatty Acid, and Antimicrobial Activity Profiling

**DOI:** 10.3390/foods15213937

**Published:** 2026-11-03

**Authors:** Alanood A. Alfaleh, Tahreer M. Alraddadi, Maha A. Althaiban, Woroud A. Alsanei, Shatha H. Alaoufi, Ebtihaj O. Alnasri

**Affiliations:** 1 Food and Nutrition Department, Faculty of Human Sciences and Design, King Abdulaziz University, P.O. Box 80200, Jeddah 21589, Saudi Arabia; aaalfaleh@kau.edu.sa (A.A.A.); walsanei@kau.edu.sa (W.A.A.); ealnassri@kau.edu.sa (E.O.A.); 2 Chemistry Department, Al-Qunfudah University College, Umm Al-Qura University, P.O. Box 1109, Makkah 21912, Saudi Arabia; tmradade@uqu.edu.sa; 3 Department of Early Childhood, College of Education, Qassim University, P.O. Box 4518, Buraidah 52571, Saudi Arabia; shatha.alaoufi@qu.edu.sa

**Keywords:** samh flour, *Mesembryanthemum cryptanthum*, phenolic compounds, phytochemical profiling, fatty acid, mineral composition, antioxidant activity, antimicrobial activity

## Abstract

Samh flour (SF), an underutilized traditional food product in Saudi Arabia, was evaluated for its nutritional composition, phytochemical profile, antioxidant capacity, mineral content, lipid constituents, polysaccharide-enriched fraction, and antimicrobial activity. SF was fractionated using hexane, ethyl acetate, butanol, and water. Proximate analysis showed that carbohydrates (63.38 g/100 g) and proteins (22.69 g/100 g) were the major nutritional components, together with low moisture and moderate fat contents. Targeted LC–MS/MS analysis showed that the ethyl acetate fraction contained the highest measured concentrations of several phenolic acids and flavonoids, particularly 3,4-dihydroxybenzoic acid, ferulic acid, naringenin, and kaempferol. This fraction also showed the lowest numerical DPPH IC_50_ value (6.22 ± 0.72 µg/mL) compared with the other SF fractions and the catechin standard (9.23 ± 1.83 µg/mL). ICP–OES analysis indicated appreciable concentrations of macroelements, including P, K, Mg, Na, and Ca, and trace elements such as Fe, Mn, and Zn, while the analyzed toxic elements were detected at low concentrations in the examined sample. General LC–MS screening of the ethyl acetate, butanol, and aqueous fractions provided tentative annotations of several metabolite classes, including amino acids, organic acids, nucleosides, phenolic derivatives, and flavonoid glycosides. GC–MS analysis of the hydrolyzed and derivatized polysaccharide-enriched fraction provided a provisional profile dominated by trimethylsilylated sugar derivatives. GC–MS screening of the methylated saponifiable fraction revealed selected peaks tentatively assigned as fatty acid methyl esters, including derivatives of palmitic, stearic, and linoleic acids, whereas the unsaponifiable fraction contained hydrocarbons and a tentatively annotated wax ester. The volatile fraction contained several unsaturated aldehydes and medium-chain hydrocarbons. Preliminary antimicrobial assays demonstrated inhibitory activity against selected bacterial and fungal strains, and MIC, MBC, and MFC endpoints were determined for the active fractions. Overall, this study provides a comprehensive chemical and biological characterization of SF and supports its potential as a functional food and a promising source of bioactive compounds.

## 1. Introduction

Samh flour (SF) is a food product derived from the seeds of samh (*Mesembryanthemum cryptanthum*). This plant is among the most well-known species thriving in the semi-arid zones of the Middle East and is considered an important cultivated plant. It is highly valued in Saudi Arabia, especially in the Al-Jouf region, as well as in Jordan, for its seeds, which are harvested after the plant dries following the rainy season [1]. Nutritionally, samh seeds are rich in amino acids, fatty acids, and carbohydrates and provide essential minerals, including sodium, calcium, potassium, magnesium, iron, zinc, manganese, and copper [2]. Phytochemically, samh seeds are recognized as a valuable source of natural products and bioactive secondary metabolites [3,4]. Studies have reported the presence of antioxidant enzymes, alkaloids, phenolic compounds, flavonoids, and tannins [5,6]. Phytochemically, samh seeds belong to the family *Aizoaceae*. Plants in this family have diverse pharmacological properties, including antihyperlipidemic, antipyretic, diuretic, antioxidant, anticancer, larvicidal, analgesic, antirheumatic, anticholera, emetic, laxative, anti-inflammatory, and antimicrobial activities [6,7,8].

Traditionally, due to their nutritional value, samh seeds are widely used in food preparation. The seeds are roasted and ground into flour, which is commonly used to prepare bread or mixed with dates and butter to produce a local dish known as Pakilla. In more recent applications, SF has been incorporated into food processing; for instance, it has been used to replace 30% of wheat flour in cookies and to substitute 3.5% soybean meal in beef patties [9]. The incorporation of 15% SF into baked goods has been associated with lower creatinine levels and improved lipid profiles, including beneficial effects on cholesterol and high- and low-density lipoprotein. Its nutritional composition includes 20.5% protein, 63.05% carbohydrates, 4.8% fats, and 10.6% dietary fiber, making it a valuable dietary component [10]. Moreover, the presence of phytosterols in SF has been linked to cholesterol-lowering effects and potential antifungal activity and has been reported to reduce triglyceride and glucose levels [11]. The seeds also contain organic acids (e.g., citric, malic, and oxalic acids) and polyols known for their health-promoting properties [12].

Recent studies have further highlighted the nutritional and technological potential of Samh seeds. Alkaltham et al. demonstrated that microwave heating combined with hot air improved protein solubility, functional properties, and pasting characteristics, suggesting opportunities for optimizing Samh processing for food applications [13]. More recently, Alanazi et al. reported differences in the phytochemical profiles of Samh populations collected from the Al-Jouf and Qassim regions of Saudi Arabia, highlighting the potential influence of geographical origin on their chemical composition [14]. These findings emphasize the importance of further investigating the compositional characteristics of Samh as a potentially valuable food ingredient.

Phytochemical characterization commonly relies on complementary analytical techniques, such as gas chromatography–mass spectrometry (GC–MS) and liquid chromatography–mass spectrometry (LC–MS), which offer high sensitivity and selectivity for detecting a broad range of plant metabolites [15]. GC–MS is particularly suitable for volatile, semi-volatile, and derivatized compounds, whereas LC–MS is more appropriate for polar, non-volatile, and thermally labile constituents [16]. These techniques offer rapid analysis, high resolution, and minimal sample requirements, making them valuable tools for profiling phytochemical constituents in plant materials [17].

Recent studies have investigated the nutritional composition, phytochemical characteristics, and antioxidant properties of Samh, including the effects of processing on its nutritional and functional attributes [4,6,18]. The present study complements these findings by providing an integrated chemical characterization of commercially obtained Samh flour using targeted phenolic analysis and exploratory LC–MS and GC–MS profiling.

Accordingly, this study aimed to (i) characterize the mineral and secondary metabolite profiles of Samh flour using ICP–MS, LC–MS/MS, and GC–MS; (ii) extract the fixed oil and determine its relative fatty acid composition through fatty acid methyl ester (FAME) analysis; (iii) extract the polysaccharide fraction and characterize its sugar-related composition following hydrolysis and derivatization; (iv) compare the antioxidant activities of different solvent fractions; and (v) investigate the antimicrobial activities of these fractions against selected bacterial and fungal pathogens using MIC, MBC/MFC, and time-kill kinetic analyses.

## 2. Materials and Methods

### 2.1. Reagents and Chemicals

High-performance liquid chromatography (HPLC)-grade solvents—including methanol, trifluoroacetic acid (TFA), acetonitrile, diethyl ether, and ethyl acetate were obtained from Fisher Scientific UK Ltd. (Loughborough, UK). Folin–Ciocalteu phenol reagent was purchased from Sigma-Aldrich (St. Louis, MO, USA), whereas sodium carbonate, potassium hexacyanoferrate, and ferric chloride were supplied by BDH Chemicals Ltd. (Poole, UK). The 1,1-diphenyl-2-picrylhydrazyl (DPPH) radical was obtained from Sigma-Aldrich. High-purity (>99%) phenolic standards used for HPLC analysis included gallic acid, caffeic acid, rutin, coumaric acid, vanillin, naringenin, quercetin, ellagic acid, 3,4-dihydroxybenzoic acid, hesperetin, cinnamic acid, methyl gallate, kaempferol, ferulic acid, syringic acid, apigenin, catechin, daidzein, chlorogenic acid, resveratrol, and rosmarinic acid. A multi-element standard solution (1000 mg/L) containing all analyzed elements was purchased from Merck (Darmstadt, Germany) for calibration by inductively coupled plasma optical emission spectrometry (ICP–OES). Analytical-grade nitric acid (HNO_3_, 65%) and hydrogen peroxide (H_2_O_2_, 30%) from Merck were used for sample digestion. Ultrapure water was generated using a Milli-Q purification system (Millipore, Merck Millipore, Molsheim, France). Nutrient agar (NA) and nutrient broth (NB) media were used for antimicrobial assays.

### 2.2. Materials and Extract Preparation

Samh seed flour (SF) was purchased as a ready-to-use traditional food product from a local market in the Al-Jouf region, Saudi Arabia. A single commercial flour sample was obtained from one source, without independent sampling from additional farms or suppliers. The flour was purchased in its prepared form and was not subjected to additional roasting or milling in the laboratory. Upon receipt, the sample was thoroughly mixed to improve homogeneity and stored frozen until laboratory analysis. The reported results therefore characterize the commercial sample investigated and should not be considered representative of all Samh flour produced in the region.

Approximately 5 g of the homogenized flour was extracted with 50 mL of 85% methanol. The mixture was sonicated for 30 min and centrifuged at 6000× *g* for 10 min. The extraction was performed three times in total on the same sample, and the resulting supernatants were pooled and filtered through a 0.45 µm PTFE membrane. The combined methanolic extract was concentrated under reduced pressure to remove methanol and then reconstituted in distilled water. The resulting aqueous suspension was successively partitioned with equal volumes of n-hexane, ethyl acetate, and n-butanol. The residual aqueous phase was retained as the water fraction. Each fraction was concentrated to dryness under reduced pressure and stored at 4 °C until subsequent chemical analyses. The 85% methanol–water mixture was selected as the initial extraction solvent because it facilitates the recovery of a broad range of polar and moderately polar phytochemicals, particularly phenolic compounds, flavonoids, and their glycosides [19]. Subsequent liquid–liquid partitioning enabled the extracted constituents to be distributed according to their relative polarity. The n-hexane fraction was expected to contain predominantly non-polar constituents, including lipids and other lipophilic compounds; the ethyl acetate fraction contained mainly compounds of intermediate polarity; the n-butanol fraction retained relatively polar constituents, including some phenolics and glycosides; and the residual aqueous fraction contained the most highly polar water-soluble compounds. This polarity-based fractionation facilitated comparison of the chemical composition and antioxidant activity of the different fractions.

### 2.3. Nutritional Analysis of Samh Flour

Nutritional analyses were performed according to Association of Official Analytical Collaboration International (AOAC) guidelines [20]. Moisture content was determined by drying the SF samples at 105 °C under controlled conditions, and the percentage was calculated from the difference between the initial and final weights. Total protein was determined by the Kjeldahl method using a nitrogen-to-protein conversion factor of 6.25. Ash content was measured by initially drying the samples at 100 °C and subsequently incinerating them in a muffle furnace at 550 °C until a white or light gray ash residue was obtained. Crude fiber content was determined using the Weende method through sequential acid hydrolysis with 1.25% H_2_SO_4_ followed by alkaline hydrolysis with 1.25% NaOH [21]. The reported carbohydrate value was consistent with calculation by difference, excluding crude fiber using the following Equation (1):

carbohydrates (%) = 100 − [moisture (%) + ash (%) + fat (%) + protein (%) + crude fiber (%)].(1)

The analyses were conducted using a Kjeldahl digestion unit and a Vapodest 20s distillation unit, together with a muffle furnace (Vulcan, WV, USA) and a drying oven (Binder, Baden-Württemberg, Germany). All measurements were performed under controlled laboratory conditions at 20 °C and 37% relative humidity.

### 2.4. LC–MS/MS Analytical Procedure

Phenolic compounds in the Samh flour (SF) fractions were analyzed according to Seraglio et al. [22], with minor modifications, using liquid chromatography–electrospray ionization–tandem mass spectrometry (LC–ESI–MS/MS). Each extract was dissolved in HPLC-grade methanol and filtered through a 0.22 µm PTFE membrane before analysis. Chromatographic separation was performed using an ExionLC™ AC system (Danaher company, DC, USA) coupled to a SCIEX Triple Quad 5500+ mass spectrometer (Singapore) equipped with an electrospray ionization source. Separation was achieved on a Poroshell 120 EC-C18 column (3.0 × 100 mm, 2.7 µm). The mobile phases consisted of solvent A (0.1% formic acid in water) and solvent B (LC-grade acetonitrile). The gradient program was as follows: 0–1 min, 8% B at 0.5 mL/min; 1–4 min, a linear increase from 8% to 15% B with the flow rate reduced to 0.4 mL/min; 4–12 min, 15–20% B at 0.4 mL/min; 12–20 min, 20–30% B at 0.4 mL/min; 20–25 min, 30–45% B at 0.4 mL/min; 25–25.01 min, 45–8% B with the flow rate increased to 0.5 mL/min; and 25.01–28 min, 8% B at 0.5 mL/min for column re-equilibration. The injection volume was 5 µL.

The mass spectrometric analyses were conducted in separate positive- and negative-ion acquisitions. Positive electrospray ionization was used for broader metabolite profiling, and the detected ions were reported predominantly as protonated molecules [M+H]+. Metabolites reported without MS/MS fragmentation (w/o MS2) were considered tentatively annotated based on their precursor ions and database matches. Targeted quantification of phenolic compounds was performed in negative electrospray ionization mode using multiple reaction monitoring (MRM). The negative-ion source parameters were as follows: curtain gas, 25 psi; collision gas, 9 psi; ion-spray voltage, −4500 V; source temperature, 500 °C; and ion-source gases 1 and 2, 50 psi each. Compound-specific precursor-to-product ion transitions (Q1 → Q3) were monitored, and the declustering potential, collision energy, and collision-cell exit potential were optimized for each transition. The precursor ions listed in the targeted MRM table corresponded to deprotonated molecules [M−H]−. Quantification was performed using external calibration curves prepared from authentic reference standards. Data acquisition and processing were conducted using SCIEX Analyst software. Accordingly, the [M+H]+ ions reported in the metabolite-profiling tables and the [M−H]− ions reported in the quantitative phenolic table originated from different acquisition modes.

### 2.5. DPPH Free Radical Scavenging Assay

The antioxidant activity of the SF extracts was evaluated using the 2,2-diphenyl-1-picrylhydrazyl (DPPH) free radical-scavenging assay, following the method described by Uddin et al. [23]. Briefly, the methanolic extracts were dissolved in 50% methanol to obtain concentrations ranging from 2.5 to 10 µg/mL. Subsequently, 40 µL of each fraction was mixed with 160 µL of 150 µM DPPH solution in methanol. After 30 min incubation in the dark at room temperature, the reduction of DPPH free radicals was measured by recording the absorbance at 517 nm. The control consisted of 160 µL of DPPH solution mixed with 40 µL of 50% methanol. All experiments were performed in triplicate, and the results were expressed as mean ± SD in Table S1. Catechin was used as the reference antioxidant and working solutions of different concentrations were prepared from a stock solution (0.1 g/100 mL methanol). The extract concentration (µg/mL) required to scavenge 50% of DPPH radicals (IC_50_) was calculated. The percentage inhibition was determined using the following Equation (2):

(2) $$\mathrm{DPPH}\;\mathrm{scavenging}\;(\%)=\frac{\mathrm{Ao}-\mathrm{As}}{\mathrm{Ao}}\times 100,$$

Here, Ao is the absorbance of the blank and As is the absorbance of the sample or standard measured at 517 nm. IC_50_ represents the concentration of the sample required to reduce 50% of the DPPH radicals.

### 2.6. Determination of Total Phenolic Content

The total phenolic content (TPC) of the individual SF extracts was determined using the Folin–Ciocalteu method [24]. Briefly, 0.1 mL of extract solution (5 mg/mL) or gallic acid standard (6.25–100 µg/mL) was mixed with 0.5 mL of 10% Folin–Ciocalteu reagent. After 15 min, 0.4 mL of sodium carbonate solution (7.5%) was added. The mixture was incubated for 2 h at room temperature, and the absorbance was measured at 760 nm using a UV spectrophotometer (Jasco V-730) against a blank without extract. TPC was expressed as milligrams of gallic acid equivalents per gram of the dry extract (mg GAE/g extract). All measurements were performed in triplicate.

### 2.7. Determination of Flavonoid Content

Total flavonoid content (TFC) of the individual SF extracts was determined using the method described by Zhishen et al. [25]. Briefly, 0.5 mL of extract solution (5 mg/mL) or rutin standard (6.25–100 µg/mL) was mixed with 0.5 mL of 2% (*w*/*v*) AlCl_3_ solution in methanol. The mixture was incubated for 15 min at room temperature, and the absorbance was measured at 430 nm using a UV spectrophotometer (V-730) (Jasco Corporation, Tokyo, Japan) against a blank. Total flavonoid content was expressed as milligrams of rutin equivalent per gram of dry extract (mg RE/g extract). All measurements were performed in triplicate.

### 2.8. Determination of Minerals

Elemental analysis was performed following closed-vessel microwave-assisted digestion using HNO_3_ and H_2_O_2_, as described by Yilmaz et al. [26] and da Silva et al. [27]. Briefly, 1.0 g of SF was accurately weighed into a digestion vessel, and 6 mL of concentrated nitric acid (HNO_3_) and 1 mL of hydrogen peroxide (H_2_O_2_) were added. The mixture was allowed to pre-digest for 30 min before microwave digestion. The vessels were then sealed and placed in a microwave digestion system (Pro-8054 Multiwave), (Anton Paar, Graz, Austria) where digestion was performed at 200 °C for 20 min. All plastic and glassware were pre-cleaned by soaking in dilute nitric acid and thoroughly rinsed with distilled water to minimize contamination. After cooling, the digested samples were quantitatively transferred into 50 mL centrifuge tubes and diluted to volume with 5% nitric acid before ICP-OES analysis. Reagent blanks containing the same reagents were prepared simultaneously. Elemental quantification was performed using an Agilent 5100S ICP-OES system. Sequential wavelength selection was applied to ensure the most sensitive analytical line for each element. Calibration standards ranging from 0.05 to 10 mg/L^−1^ were used. Sample solutions and blanks were introduced into the plasma to determine essential elements (K, Mg, Na, Mn, Zn, Fe, Co, Ni, Cu, P, Ca, and Cr), nonessential elements (B, Al, and Sr), and heavy metals (Cd, Pb, Hg, and As).

### 2.9. Determination of Polysaccharides

Polysaccharides from SF were extracted and purified following the methods described by Luo et al. [28] with minor modifications. Briefly, 10 g of SF powder was defatted with ethanol at 80 °C and then extracted with 100 mL of deionized water at room temperature for 3 h; this extraction was repeated three times. After filtration, the recovered plant residue was further extracted with hot water in an 80 °C water bath for 60 min, also repeated three times. The hot-water extracts were filtered, combined, and concentrated under reduced pressure. Polysaccharides were precipitated by adding four volumes of absolute ethanol, followed by centrifugation at 4000 rpm for 15 min. The precipitate was deproteinized using Sevag reagent (chloroform, 4:1, *v*/*v*), dried at 40 °C, and weighed, giving an extraction yield of 3.2%. For hydrolysis, 10 mg of the polysaccharide powder was mixed with 10 mL of 2 M trifluoroacetic acid in sealed tubes and heated at 120 °C for 2 h. After cooling, a 100 µL aliquot of the hydrolysate was transferred to a 1 mL vial and freeze-dried. The dried residue was dissolved in 100 µL of pyridine, mixed with 100 µL of N,O-bis(trimethylsilyl)trifluoroacetamide, and incubated at 60 °C for 60 min. The derivatized hydrolysate was transferred to a 200 µL insert and analyzed using a Thermo Trace 1300 gas chromatograph coupled to a Thermo ISQ 7000 mass spectrometer. Separation was performed on a TG-5MS fused-silica capillary column (30 m × 0.32 mm i.d., 0.25 µm film thickness) using helium at a flow rate of 1 mL/min and a pressure of 13 psi. The oven temperature was initially maintained at 80 °C for 5 min, increased to 300 °C at 5 °C/min, and held at 300 °C for 5 min. The ion source temperature was 180 °C, the electron-ionization energy was 70 eV, and the injection volume was 1 µL. Detected components were tentatively assigned by comparing their EI mass spectra with the Wiley and NIST libraries, and their relative abundances were estimated from normalized peak-area percentages.

### 2.10. Determination of Fixed and Volatile Oils

#### 2.10.1. Extraction and Determination of Fixed Oil

Extraction, saponification, and preparation of fatty acid methyl esters (FAMEs) were performed according to Ichihara et al. [29]. Briefly, 23 g of SF was placed in a cellulose thimble and extracted with 500 mL of hexane using a Soxhlet apparatus at 60 °C for 6 h. The extract was concentrated under reduced pressure at 40 °C, yielding 1.23 g of fixed oil, corresponding to 5.35% of the original flour weight. An aliquot of the oil (0.5 g) was refluxed with 40 mL of 10% alcoholic KOH prepared in 95% ethanol for 4 h. After cooling, the unsaponifiable fraction was extracted with n-hexane, dried over anhydrous sodium sulfate, filtered, and concentrated. The remaining alcoholic layer was acidified with 40 mL of 20% HCl to release the free fatty acids, which were extracted with n-hexane, dried over anhydrous sodium sulfate, filtered, and evaporated. The isolated fatty acids were refluxed with 50 mL of 2% H_2_SO_4_ in anhydrous methanol for 3 h. After cooling, the resulting FAMEs were extracted with n-hexane, dried over anhydrous sodium sulfate, filtered, and concentrated under reduced pressure before GC–MS analysis.

The detected FAMEs were tentatively identified by comparing their EI mass spectra with those available in the Wiley and NIST spectral libraries. Retention times were recorded for chromatographic reporting but were not used alone for compound confirmation. Fatty acid composition was expressed as relative peak-area percentage, calculated by normalizing the area of each identified FAME peak to the total area of the detected FAME peaks. Accordingly, these values represent the relative fatty acid composition of the extracted oil and not absolute concentrations or quantities relative to the original flour weight.

#### 2.10.2. Extraction and Determination of Volatile Oil

Volatile oil was extracted from 50 g of SF mixed with 800 mL of distilled water in a round-bottom flask. A Clevenger-type apparatus designed for oils lighter than water was assembled, and the mixture was heated to boiling on a heating mantle for 3 h. The condensed oil–water mixture was collected, and the separated essential oil layer (15 μL of yellow oil) was recovered, dried over anhydrous sodium sulfate, and diluted with hexane before analysis [30].

#### 2.10.3. Gas Chromatography–Mass Spectrometry

The volatile oil obtained from SF was diluted in hexane (1 μL per 1 mL hexane) and analyzed using a Thermo Trace 1300 GC equipped with an AI 1310 autosampler and coupled to a Thermo ISQ 7000 mass spectrometer. Separation was performed on a fused-silica capillary column (30 m × 0.25 mm × 0.25 µm; TG-5MS stationary phase). Helium was used as the carrier gas at a flow rate of 1 mL/min and a pressure of 13 psi. The oven temperature program was set to 60 °C (held for 5 min), then ramped to 280 °C at 5 °C/min, followed by a final hold at 280 °C for 5 min. The ion source temperature was maintained at 250 °C, the transfer line at 290 °C, and the ionization voltage at 70 eV, with a scan range of *m*/*z* 40–800. A 1 µL sample was injected in splitless mode. Compounds were identified by comparing their mass spectra and retention times with those in the Wiley and NIST libraries [31].

### 2.11. Antimicrobial Activity Assay

Antimicrobial activity was evaluated using nutrient agar (NA) and nutrient broth (NB) media prepared according to standard microbiological procedures. The NA medium contained yeast extract, peptone, meat extract, sodium chloride, and agar, with a final pH of 7.4 ± 0.2, whereas the NB medium contained the same components without agar. The ethyl acetate, butanol, and aqueous fractions of SF were separately dissolved in DMSO at a concentration of 50 mg/mL for the initial antimicrobial screening. Antimicrobial activity was evaluated against Gram-negative bacteria (*Escherichia coli* ATCC 25922 and *Pseudomonas aeruginosa* ATCC 27853), Gram-positive bacteria (*Bacillus cereus* ATCC 6629, *Bacillus subtilis* ATCC 6633, and *Staphylococcus aureus* ATCC 6538), and fungi (*Candida albicans* ATCC 10231, *Aspergillus niger* ATCC 16888, and a local isolate of *Aspergillus flavus*).

Qualitative antimicrobial screening was performed using the well-diffusion method described by Klancnik et al. [32]. Inoculate of the tested microorganisms were prepared from fresh cultures and adjusted to a turbidity equivalent to a 0.5 McFarland standard (1.5 × 10^8^ CFU/mL). Subsequently, 25 µL of each standardized inoculum was transferred into sterile Petri dishes containing 50 mL of solidified nutrient agar. Wells measuring 0.6 cm in diameter were prepared using a sterile cork borer. Each well was filled with 75 µL of the corresponding SF fraction at 50 mg/mL, equivalent to 3.75 mg of extract per well. The inoculated plates were refrigerated for 1 h to allow diffusion of the extracts into the medium. Plates inoculated with bacterial strains and *C. albicans* were incubated at 37 °C for 24 h, whereas plates inoculated with *A. niger* and *A. flavus* were incubated at 29 °C for 120 h. Antimicrobial activity was evaluated by measuring the inhibition-zone diameter in millimeters. Measurements were performed in duplicate. Gentamicin (10 µg/disc) and miconazole (50 µg/disc) were used as positive controls for bacterial and fungal strains, respectively.

The ethyl acetate and butanol fractions, which showed activity in the well-diffusion assay, were further evaluated for minimum inhibitory concentration (MIC) using the broth microdilution method described by Bona et al. [33]. Serial dilutions of each fraction were prepared in NB in sterile 96-well microtiter plates over a concentration range covering the tested extract concentrations. The concentrations were expressed as the final extract concentrations in the assay wells. A standardized microbial inoculum was added to each extract dilution, and the plates were incubated at 37 °C for 24 h. Before absorbance measurement, the plates were shaken for 20 s and allowed to stand briefly. Microbial growth was assessed by measuring absorbance at 600 nm using a microplate reader (Bio-Rad Laboratories Inc., Model 3350, Hercules, CA, USA). The MIC was defined as the lowest final extract concentration showing no detectable microbial growth, as indicated by an absorbance comparable to that of the corresponding blank.

Following MIC determination, aliquots from wells showing complete inhibition of visible growth at the MIC and higher concentrations were subcultured onto extract-free agar plates. The plates were incubated at 37 °C for 24 h. The minimum bactericidal concentration (MBC) was defined as the lowest extract concentration that produced no visible bacterial colonies after subculture. For *C. albicans*, the corresponding fungicidal endpoint was reported as the minimum fungicidal concentration (MFC). The MBC/MIC and MFC/MIC ratios were calculated to further characterize the antimicrobial activity of the tested fractions.

### 2.12. Statistical Analysis

Descriptive statistical analysis was performed using IBM SPSS Statistics, version 29.0 (IBM Corp., Armonk, NY, USA). For assays performed in triplicate, results were expressed as mean ± standard deviation (SD; *n* = 3 technical replicates). Antimicrobial inhibition-zone measurements obtained in duplicate were reported individually, while minimum inhibitory concentration (MIC), minimum bactericidal concentration (MBC), and minimum fungicidal concentration (MFC) results were presented as experimentally determined endpoints. LC–MS/MS calibration curves were evaluated using linear regression, whereas GC–MS results were expressed as relative peak-area percentages. No inferential statistical tests were performed to compare sample fractions or establish population-level differences.

## 3. Results

### 3.1. Nutritional Analysis

The compositional results presented in Table 1 indicate that the SF sample contains a high proportion of total solids (96.57 g/100 g), reflecting low moisture content and suggesting good storage stability. The ash content (3.35 g/100 g) represents the total mineral content and falls within the expected range for plant-based materials. The fat content (5.95 g/100 g) was moderate and contributed to the caloric value and functional properties of the sample. Protein content was 22.69 g/100 g, indicating a nutritionally significant level and suggesting that SF may serve as a valuable dietary protein source. The crude fiber content was relatively low (1.20 g/100 g). Carbohydrates calculated by difference, excluding crude fiber, accounted for the largest proportion (63.38 g/100 g), indicating that carbohydrates were the predominant macronutrient in the sample. Overall, the results indicate a nutrient-dense composition characterized by high carbohydrate and protein content and low moisture levels.

### 3.2. DPPH Assay

**Figure 1:**
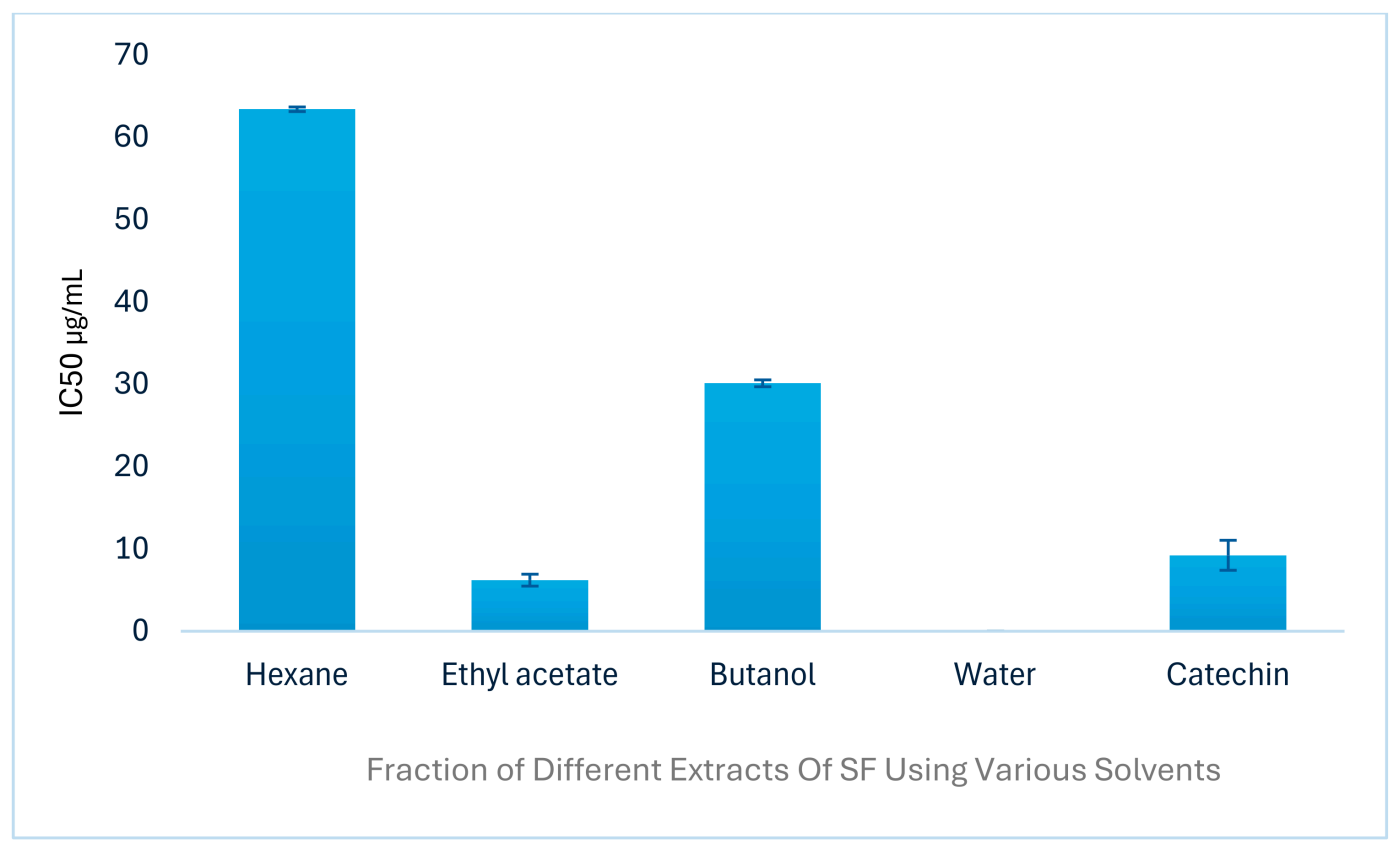
IC_50_ Values of SF Extract Fractions in the DPPH Radical-Scavenging Assay. Error bars represent standard deviations (SD).

Figure 1 presents the DPPH radical-scavenging activity of the SF fractions obtained using butanol, ethyl acetate, water, and hexane, expressed as IC_50_ values. A lower IC_50_ value indicates greater radical-scavenging activity. Among the tested fractions, the ethyl acetate fraction showed the lowest numerical IC_50_ value (6.22 ± 0.72 µg/mL), which was also numerically lower than that of the catechin standard (9.23 ± 1.83 µg/mL). The butanol fraction showed an IC_50_ value of 30.12 ± 0.42 µg/mL, whereas the hexane fraction showed a higher IC_50_ value of 63.40 ± 0.28 µg/mL. An IC_50_ value could not be determined for the aqueous fraction under the tested conditions. Overall, the ethyl acetate fraction exhibited the lowest numerical IC_50_ value among the SF fractions, which may be associated with its higher phenolic and flavonoid content.

### 3.3. Total Phenolic and Total Flavonoid Contents

The TPC and TFC of the SF extracts varied according to the extraction solvent (Table 2). The reported TPC and TFC values represent assay-based estimates expressed as gallic acid and rutin equivalents, respectively, rather than absolute concentrations of all phenolic and flavonoid compounds. The ethyl acetate extract exhibited the highest levels of phenolics and flavonoids, with mean values of 33.61 mg GAE/g extract for TPC and 21.90 mg RE/g extract for TFC. The butanol extract displayed moderate levels of phenolics (19.56 mg GAE/g) and flavonoids (5.11 mg RE/g). In contrast, the hexane extract contained comparatively low amounts of phenolics (7.20 mg GAE/g) and flavonoids (3.60 mg rutin/g). The aqueous extract showed the lowest TPC (5.75 mg GAE/g) and no detectable flavonoids, indicating limited extraction of these compounds from the matrix. Collectively, these results demonstrate that ethyl acetate is the most effective solvent for extracting phenolic and flavonoid compounds from SF, suggesting that this fraction may possess the highest antioxidant-related phytochemical activity.

### 3.4. ICP Analysis

Table 3 presents the mineral composition of the SF sample determined by ICP analysis. The results show that the sample is rich in several nutritionally important macroelements, with phosphorus (3000 mg/kg), potassium (1700 mg/kg), magnesium (975 mg/kg), sodium (700 mg/kg), and calcium (300 mg/kg) as the predominant elements. These concentrations indicate a mineral-dense composition that may support essential physiological and biochemical functions. Boron was also present at a notable level (64 mg/kg), consistent with its role in plant structural integrity and metabolic processes.

Among the essential trace elements, iron (48 mg/kg), manganese (75 mg/kg), zinc (15 mg/kg), and copper (10 mg/kg) were detected in appreciable amounts, indicating the potential contribution of the seeds to micronutrient intake. Other elements, including nickel (1.25 mg/kg), cobalt (0.05 mg/kg), chromium (0.05 mg/kg), and selenium (0.05 mg/kg), were detected at lower concentrations, which is typical for plant-derived materials. Aluminum (6.25 mg/kg), although not considered an essential nutrient, was present within the range commonly reported in natural plant tissues.

The concentrations of heavy metals, including cadmium (<0.05 mg/kg), arsenic (<0.05 mg/kg), lead (<1 mg/kg), and mercury (<0.05 mg/kg) were below the corresponding analytical reporting limits in the examined sample. Overall, the elemental profile demonstrates that the seeds contain high levels of beneficial macro- and microelements, while the analyzed toxic elements were below the corresponding analytical reporting limits.

### 3.5. LC–MS/MS Analysis

#### Quantification of Phenolic Acids and Flavonoids

The LC–MS/MS calibration parameters, including regression equations, coefficients of determination (R^2^), relative standard deviations (RSD), limits of detection (LOD), and limits of quantification (LOQ), are summarized in Table 4. Quantitative LC–MS/MS analysis revealed marked differences in the concentrations of phenolic compounds among the solvent fractions of SF extract, as presented in Table 5.

The ethyl acetate fraction consistently exhibited the highest concentrations for most detected compounds, indicating its superior efficiency in extracting medium-polarity phenolics. Compounds such as 3,4-dihydroxybenzoic acid (2732.14 µg/g), ferulic acid (410.36 µg/g), coumaric acid (102.09 µg/g), naringenin (248.39 µg/g), kaempferol (225.54 µg/g), and quercetin (67.91 µg/g) were highly enriched in this fraction. Other phenolic acids, including gallic acid (12.36 µg/g), caffeic acid (80.63 µg/g), vanillin (77.66 µg/g), cinnamic acid (111.66 µg/g), and syringic acid (69.02 µg/g), were also present at relatively high levels in the ethyl acetate extract, demonstrating its broad extraction capacity.

The butanol fraction contained moderate levels of several phenolics. The most abundant compounds were naringenin (305.37 µg/g), followed by vanillin (55.80 µg/g), cinnamic acid (51.70 µg/g), 3,4-dihydroxybenzoic acid (30.20 µg/g), and kaempferol (25.78 µg/g). Additional compounds, including rutin, chlorogenic acid, rosmarinic acid, and coumaric acid, were detected at lower concentrations than those observed in the ethyl acetate fraction, indicating lower extraction efficiency and a narrower range of phenolics.

The aqueous fraction exhibited the lowest concentrations overall, with most compounds either absent or detected only at trace levels. Notable exceptions include rutin (7.37 µg/g), 3,4-dihydroxybenzoic acid (35.5 µg/g), chlorogenic acid (2.93 µg/g), and quercetin (1.12 µg/g), suggesting that only a limited number of hydrophilic phenolics partitioned into the aqueous phase. Many compounds—such as caffeic acid, naringenin, vanillin, cinnamic acid, kaempferol, apigenin, and ferulic acid—were undetectable in this fraction, indicating their preferential association with solvents of intermediate polarity.

Overall, the results demonstrate that ethyl acetate was the most efficient solvent for extracting phenolic acids and flavonoids, followed by butanol, whereas water exhibited minimal extraction efficiency. This pattern is consistent with the polarity characteristics of phenolic compounds, most of which possess moderate polarity and therefore dissolve more readily in ethyl acetate. The pronounced enrichment of compounds such as ferulic acid, 3,4-dihydroxybenzoic acid, naringenin, and kaempferol in the ethyl acetate fraction further suggests that this fraction has the highest antioxidant and bioactive potential.

### 3.6. LC–MS–Based Metabolite Profiling of SF Extract Fractions

LC–MS profiling of the ethyl acetate, butanol, and aqueous extracts revealed distinct metabolic fingerprints consistent with solvent polarity and phytochemical solubility Table 6, Table 7 and Table 8.

### 3.7. GC–MS Analysis of Polysaccharides

Table 9 shows that the GC–MS profile of the polysaccharide extract following acid hydrolysis and trimethylsilyl derivatization was dominated by TMS derivatives of monosaccharides and related sugar compounds. The most prominent peaks were tentatively assigned to pentakis-O-trimethylsilyl hexopyranoses and TMS derivatives of glucose, talose, and mannose, indicating the presence of neutral sugar-related components in the hydrolyzed extract. Signals assigned to trimethylsilylated melibiose and ribose derivatives suggest the presence of disaccharide- and pentose-related components. Minor phenolic derivatives, including dimethoxy phenols, and organic acid derivatives, such as TMS hexenoate, were also detected, indicating the presence of noncarbohydrate compounds co-extracted with the carbohydrate fraction.

### 3.8. Composition of Fixed Oil

The GC–MS profile presented in Table 10, obtained after saponification and methanolysis, revealed several peaks tentatively annotated as FAMEs. The chromatographic profile included medium- and long-chain saturated and unsaturated fatty acids, primarily detected as methyl esters. Methyl linoleate was the most abundant selected FAME-related peak (19.81%), followed by methyl palmitate (5.73%) and methyl stearate (1.39%). Other methyl esters of tetradecanoic, pentadecanoic, heptadecanoic, and eicosanoic acids were detected at lower relative peak areas. The detection of branched-chain and cyclopropane derivatives suggests additional diversity within the lipid fraction; however, these annotations remain tentative. A phthalate-related peak detected at a later retention time was considered a probable plasticizer contaminant and was excluded from the fatty acid interpretation.

In contrast, the unsaponifiable fraction (Table 11) exhibited a distinct profile containing hydrocarbons tentatively annotated as tetradecane and docosane. The prominent peak detected at RT 51.59 min produced a low-confidence ester-related library match (SI = 635) and was therefore retained as an unidentified high-boiling component. This match may reflect incomplete saponification, carryover during fractionation, or library misidentification.

Overall, these findings provide a preliminary characterization of the lipid fractions. The methylated saponifiable fraction contained several tentatively annotated FAME-related peaks, whereas the unsaponifiable fraction contained hydrocarbons and unresolved high-boiling components.

### 3.9. Volatile Oil

Table 12 illustrates the GC–MS analysis of the volatile fraction, which revealed a complex mixture of aldehydes, hydrocarbons, ketones, and esters. The chromatographic profile was dominated by unsaturated aldehydes, including (E)-2-decenal, 2,4-decadienal (E,E), and 2-undecenal, which collectively accounted for a major portion of the volatile profile. Medium- to long-chain alkanes—including decane, undecane, dodecane, tetradecane, and nonadecane—were also consistently detected as background constituents. Minor amounts of oxygenated compounds, including 1-dodecanol, 2-nonenal, and 2-pentadecanone, were observed, suggesting the presence of lipid-derived volatiles formed through the oxidative cleavage of higher fatty acids.

### 3.10. Antimicrobial Study

The antimicrobial evaluation showed that the tested SF fractions exhibited inhibitory activity against the microorganisms examined. In the well-diffusion assay (Table 13), the ethyl acetate and butanol fractions were tested at 50 mg/mL, using 75 µL per well (3.75 mg extract/well). The extracts produced measurable inhibition zones against Gram-negative and Gram-positive bacteria. E. coli showed inhibition-zone diameters of 21–22 mm. Activity was also observed against Pseudomonas aeruginosa, while moderate inhibition was observed against Bacillus species and Staphylococcus aureus. Antifungal activity was evident, particularly against Aspergillus flavus. Although the extract produced larger inhibition zones than the reference antifungal disc, differences in the applied amounts and diffusion characteristics preclude a direct comparison of potency. The MIC results (Table 14) provided additional evidence of antimicrobial activity. The butanol fraction showed lower MIC values, particularly against Staphylococcus aureus, Pseudomonas aeruginosa, and Candida albicans. The MBC and MFC results (Table 15) indicated bactericidal and fungicidal activity under the tested conditions, with the butanol fraction requiring lower concentrations to produce no visible growth after subculture onto extract-free agar. Overall, these findings provide preliminary evidence of antimicrobial activity against the tested bacterial and fungal strains. The MBC/MIC and MFC/MIC ratios are presented in Table 16. The reported MBC/MIC ratios of ≤4 are consistent with bactericidal activity under the assay conditions. Photographs of the antibacterial and antifungal assays are provided in the Supplementary Materials (Figures S1–S13).

## 4. Discussion

### 4.1. Nutritional Profile

The compositional analysis of SF in this study indicates a carbohydrate-rich profile with appreciable protein content, supporting its potential for further investigation in food applications. The low moisture content and high total solids (>96%) characterize the composition of the analyzed flour batch; however, storage stability requires further evaluation. The protein content (22.7%) is relatively high and consistent with previous reports, placing SF among high-protein foods such as lentils, corn, and rice [34]. SF also contains a significant proportion of carbohydrates (63.4%), confirming its role as a major energy source. Although the fat content is moderate (approximately 6%), it contributes to the overall nutritional value and functional properties of the flour in food formulations [12]. A notable difference from earlier reports concerns the crude fiber content, which was lower in the present study (1.2%) than the value of approximately 10% reported by Alrugaie et al. [10]. This variation may be attributed to differences in SF or measurement methods. The ash content (3.35%) reflects the inorganic residue of the sample after incineration, while its mineral composition was further characterized by ICP–OES. Previous studies have also investigated the mineral composition of samh seeds [35]. Overall, SF presents a balanced nutritional composition characterized by high protein and carbohydrate levels, low moisture content, and moderate levels of fat and minerals, supporting its potential as a stable and nutritionally valuable food ingredient.

From a food industry perspective, SF may be explored as a partial ingredient in composite flours for bakery products, considering its nutritional composition and traditional food uses [10]. However, its effects on processing properties, product texture, sensory quality, and storage stability require further investigation. Consumer acceptance may depend on taste, texture, and appearance, while broader social acceptance may be influenced by cultural familiarity and awareness of Samh as a traditional food. Therefore, food formulation trials and consumer acceptance studies are needed to evaluate its practical applicability.

### 4.2. Antioxidant Activity

The antioxidant activity of SF was evaluated using the DPPH radical scavenging assay, which indicated in vitro radical-scavenging activity in the tested fractions. This activity is likely associated with the presence of phytoconstituents, including ascorbic acid, phenolics, flavonoids, and glutathione, which are known to play important roles in protecting biological systems from oxidative damage [19]. The combined phytochemical and antioxidant analyses demonstrate that SF contains exhibited the highest measured phenolic and flavonoid contents in the ethyl acetate fraction. The ethyl acetate fraction exhibited the highest measured TPC and TFC values and the lowest reported IC_50_, suggesting a descriptive association between phenolic composition and in vitro radical-scavenging activity. In this study, antioxidant activity was assessed for SF extracts prepared with solvents of different polarity (ethyl acetate, butanol, hexane, and water). The ethyl acetate fraction exhibited the lowest reported IC_50_ (6.22 ± 0.72 µg/mL) among the tested SF fractions, which was numerically lower than that of the catechin standard (9.23 ± 1.83 µg/mL). This result suggests that medium-polar phenolic compounds are major contributors to the antioxidant activity. The butanol extract showed moderate activity, whereas the hexane extract showed weak activity. In contrast, an IC_50_ value could not be determined for the aqueous fraction under the tested conditions. These findings highlight the influence of solvent polarity on the extraction of antioxidant compounds and indicate that the ethyl acetate fraction is particularly enriched in phenolic constituents. Previous studies have similarly reported strong antioxidant potential in SF, attributing this activity to the presence of flavonoids, tannins, phenolic compounds, and other antioxidants, such as ascorbic acid and glutathione [4]. Overall, these findings provide further insight into the phytochemical composition of SF and support its potential use as a natural source of antioxidant compounds. The variation in TPC and TFC values among extracts emphasizes the importance of solvent selection in recovering bioactive compounds. Consistent with these findings, ethyl acetate appears to be the most effective solvent for extracting phenolic and flavonoid constituents from SF. These insights suggest that optimized extraction strategies may enhance the recovery of antioxidant phytochemicals [18].

### 4.3. Mineral Profile

The concentrations of the analyzed toxic elements were below the applicable reference limits considered in this study FAO/WHO [36]. These findings provide preliminary information on the elemental composition of the analyzed SF batch, although a comprehensive food safety assessment would require further investigation. The detected concentration of lead (1 mg/kg) is below the commonly accepted maximum limit for plant seeds and herbal materials (≤2 mg/kg), whereas cadmium (0.05 mg/kg) and arsenic (0.05 mg/kg) are also well below their recommended thresholds (≤0.3 mg/kg for Cd and ≤1 mg/kg for As). These results suggest that the SF sample is free from significant heavy metal contamination and may be considered safe for potential dietary or nutraceutical use. In contrast, the concentrations of beneficial macro elements including potassium (1700 mg/kg), magnesium (975 mg/kg), sodium (700 mg/kg), and calcium (300 mg/kg) are relatively high, indicating a nutritionally rich mineral composition. Essential trace elements–such as iron (48 mg/kg), manganese (75 mg/kg), zinc (15 mg/kg), and copper (10 mg/kg)—were also present at physiologically relevant levels, contributing to the micronutrient value of SF. These findings are consistent with previous reports by Al-jassir et al. [1]. Overall, the mineral composition of the analyzed SF batch showed appreciable levels of essential macro- and microelements, while the investigated toxic elements were reported to be below their respective analytical reporting limits. These findings highlight the potential nutritional value of SF and provide a basis for further evaluation of its suitability as a food ingredient.

### 4.4. Integration of Chemical Composition and Antioxidant Activity

The LC–MS/MS results indicated differences in the distribution of phenolic constituents among the investigated solvent fractions, which may be associated with their polarity. Among the investigated fractions, the ethyl acetate extract exhibited the highest phenolic concentration and chemical diversity, indicating its effectiveness for extracting medium-polarity phenolic acids and flavonoid aglycones. This behavior can be attributed to the intermediate dielectric constant of ethyl acetate, which facilitates the partitioning of phenolic compounds containing aromatic rings with limited hydroxyl substitution [37].

The enrichment of 3,4-dihydroxybenzoic acid, ferulic acid, coumaric acid, and major flavonoids such as quercetin and kaempferol in this fraction is particularly significant. Several of these phenolic compounds possess structural features associated with radical-scavenging activity, including phenolic hydroxyl groups and conjugated aromatic systems. Such structural features promote resonance stabilization of phenoxyl radicals, which may contribute to the observed DPPH radical-scavenging activity. Therefore, the lower reported IC_50_ of the ethyl acetate fraction may be associated with its measured phenolic composition, although the contributions of individual compounds were not directly evaluated [38].

In contrast, the butanol fraction exhibited a different phenolic profile, including naringenin, which was measured at a higher concentration in this fraction than in the ethyl acetate fraction. Glycosylation generally reduces antioxidant activity compared with aglycone forms because steric hindrance limits the accessibility of hydroxyl groups involved in radical scavenging. This structural difference may explain the moderate antioxidant activity observed in the butanol fraction, despite the presence of measurable phenolic levels. Exploratory LC–MS profiling also provided tentative annotations of nitrogen-containing metabolites and vitamin-related compounds in this fraction, although their contributions to antioxidant activity were not established [39].

The aqueous fraction showed tentative LC–MS explanation of several polar metabolites, including nucleosides, amino acid derivatives, and sugar phosphates. Targeted LC–MS/MS analysis detected rutin and selected phenolic acids in the aqueous fraction, although the overall quantified phenolic profile differed from that of the ethyl acetate fraction. Water generally extracts primary metabolites involved in cellular metabolism rather than secondary phenolic antioxidants, consistent with the weak antioxidant response observed experimentally [40].

The ethyl acetate fraction exhibited the highest measured TPC and TFC values and the lowest reported IC_50_ among the investigated SF fractions. This descriptive pattern suggests a possible association between phenolic composition and DPPH radical-scavenging activity, although no formal correlation analysis was performed. Similar solvent-dependent extraction patterns have been reported for other plant matrices, where intermediate-polarity solvents maximize the recovery of antioxidant phenolics [41,42].

Collectively, these findings suggest that solvent-based fractionation influenced the distribution of measured phenolic compounds in the investigated SF batch. The ethyl acetate fraction exhibited the highest measured TPC and TFC values and higher concentrations of most quantified phenolic compounds. In contrast, the butanol and aqueous fractions exhibited different phytochemical profiles, while the IC_50_ of the aqueous fraction remained undetermined under the tested conditions.

However, the observed antioxidant activity cannot be attributed solely to the quantified phenolic compounds. Other constituents and potential additive, synergistic, or antagonistic interactions among extract components may also contribute to the measured antioxidant activity, although these effects were not directly investigated.

### 4.5. GC–MS Analysis

GC–MS analysis of the saponified fraction several peaks tentatively explained as fatty acid methyl esters (FAMEs), including long-chain saturated and unsaturated fatty acid derivatives [43]. Among the reported FAME-related peaks, methyl linoleate exhibited the highest relative peak area (19.81%), whereas the peak tentatively assigned to methyl palmitoleate accounted for only 0.28%. The presence of these unsaturated fatty acid derivatives may be of nutritional interest, although their absolute concentrations and nutritional contributions were not determined [44].

The volatile GC–MS profile included peaks tentatively assigned to 2,4-decadienal and 2-undecenal, which have been reported as volatile compounds associated with the oxidation of linoleic and oleic acids, respectively [45]. These aldehydes may contribute to aroma formation; however, their detection alone does not establish oxidative deterioration in the investigated SF sample.

Minor amounts of odd-chain and branched-chain fatty acids were also detected. Such compounds are often associated with microbial interactions or specialized lipid biosynthetic pathways and may reflect the biological origin of the material rather than contamination [37]. Cyclopropane derivatives likely arise from structural rearrangements that occur during saponification or analytical processing [43].

From a functional perspective, the coexistence of unsaturated fatty acids and lipid-derived volatile compounds suggests that this fraction may play a dual role by providing nutritionally relevant lipids while contributing to flavor characteristics. Nevertheless, the detection of oxidation markers underscores the need to consider oxidative stability when evaluating potential food applications.

### 4.6. Antimicrobial Evaluation

The SF extracts exhibited substantial antimicrobial activity against both gram-negative and gram-positive bacteria. The highest sensitivity was observed for *E. coli* with inhibition zones of 21–22 mm, followed by *Pseudomonas aeruginosa* (17 mm), *Staphylococcus aureus* (15–17 mm), and *Bacillus subtilis* (14 mm). In contrast, a study by Ibtissen et al. [46] reported that the ethanolic extract of *M. cryptanthum* showed relatively weak antibacterial activity, with inhibition zones of 7.2 mm, 5.1 mm, 6.2 mm, and 0 mm against *Pseudomonas aeruginosa*, *Staphylococcus aureus*, *Bacillus subtilis*, and *E. coli*, respectively. Compared with these results, the antimicrobial activity observed for SF in the present study appears considerably stronger, indicating possible differences in phytochemical composition among *Mesembryanthemum* species. The MIC and MBC results obtained in this study further support the antibacterial and antifungal activities of SF extracts. A 2026 comparison of Samh populations from Al-Jouf and Qassim also reported regional differences in phytochemical profiles and antimicrobial activity [14], indicating that source-related variation may contribute to differences among studies.

Quantitative LC–MS/MS analysis revealed marked differences in the concentrations of phenolic compounds among the SF solvent fractions Table 5. The ethyl acetate fraction contained relatively high concentrations of several phenolic compounds, particularly 3,4-dihydroxybenzoic acid, ferulic acid, kaempferol, and quercetin, whereas naringenin was more abundant in the butanol fraction than in the ethyl acetate fraction. These compounds may contribute to the antibacterial activity of SF extracts through different mechanisms.

Previous studies have demonstrated that the antibacterial effects of phenolic compounds depend on their chemical structures, concentrations, and interactions with bacterial cells [47]. One proposed mechanism involves disruption of bacterial membrane integrity and alterations in membrane permeability, potentially resulting in leakage of intracellular constituents [48,49]. Borges et al. [50] demonstrated that ferulic acid can alter bacterial membrane properties and permeability in several pathogenic bacteria, including *E. coli*, *P. aeruginosa*, and *S. aureus*. Similarly, Wang et al. [51] reported that naringenin influenced membrane fluidity, fatty acid composition, and the expression of genes associated with fatty acid biosynthesis in *E. coli* and *S. aureus*. Quercetin has also been shown to modify bacterial membrane organization and fluidity [52]. These findings suggest that membrane-related effects may represent one possible mechanism contributing to the antibacterial activity observed in SF extracts.

In addition to membrane disruption, certain phenolic compounds may exert antibacterial effects by interfering with essential bacterial enzymes and nucleic acid synthesis [53]. Zhang et al. [54] demonstrated that flavonoids, including quercetin, competitively inhibit β-hydroxyacyl-acyl carrier protein dehydratase (HpFabZ), an enzyme involved in fatty acid biosynthesis in *Helicobacter pylori*. Furthermore, Plaper et al. [55] demonstrated that quercetin inhibits bacterial DNA gyrase in *E. coli*, potentially interfering with bacterial DNA replication. Mori et al. [56] also reported that certain flavonoids inhibited DNA synthesis in *Proteus vulgaris*, suggesting that interactions with nucleic acids may contribute to their antibacterial effects. Although these findings were obtained using individual phenolic compounds and different bacterial species, they provide possible mechanistic explanations for the antibacterial activity of phenolic-rich plant extracts.

Overall, the antibacterial activity observed in the present study may be partly associated with the phenolic constituents identified by LC–MS/MS analysis, particularly ferulic acid, naringenin, and quercetin. However, differences in phenolic concentrations among solvent fractions do not establish the contribution of individual compounds to the observed antibacterial effects. Other constituents and possible additive, synergistic, or antagonistic interactions may also contribute to this activity. Furthermore, although the present study demonstrated antibacterial activity, the underlying cellular and molecular mechanisms were not directly investigated. Further studies involving membrane integrity assays, enzyme inhibition assessments, and evaluation of isolated compounds are needed to clarify the mechanisms responsible for the antibacterial activity of SF extracts.

### 4.7. Food Industry Applications and Acceptance Considerations

The measured protein (22.69 g/100 g) and carbohydrate (63.38 g/100 g) contents, together with SF established traditional use [4], support exploring the flour as a partial ingredient in composite flours for traditional baked goods, bread, and biscuits. One previous study examined the nutritional and sensory attributes of cookies made with SF and date powder [10], while another evaluated the partial replacement of wheat flour with SF in bread and cookies [57]. More recently, SF was incorporated into date-based high-protein energy balls, with nutritional, digestibility, glycemic, and sensory properties assessed [58]. These findings point to potential product-development opportunities.

As a non-wheat seed flour, SF may affect gluten-network development, dough handling, product volume, and texture when incorporated into wheat-based formulations. Recent work has reported that microwave–hot-air treatment can modify selected functional and pasting properties of Samh seeds [13], Further studies are needed to evaluate the suitability of SF for food applications, including its functional properties, optimal inclusion levels, and the effects of processing and storage on product quality and stability. Consumer acceptance should also be assessed, considering sensory attributes, price, availability, and cultural familiarity.

Previous sensory evaluations of Samh-containing products reported different responses across product types and formulations [10,57,58]. Future studies should conduct product-specific sensory panels and consumer surveys and assess the food safety and shelf life of Samh-containing products before wider commercialization.

### 4.8. Study Limitations

This study provides valuable insights into the nutritional composition and bioactive profile of Samh flour. However, the findings are based on a single commercial batch, and antioxidant activity was assessed using an in vitro DPPH assay. Future studies involving samples from different sources and additional biological and food-based evaluations would further strengthen and expand these findings.

## 5. Conclusions

This study provides an integrated nutritional, phytochemical, analytical, and preliminary biological evaluation of SF. The findings indicate that SF is nutritionally valuable and contains proteins, carbohydrates, essential minerals, and several phytochemical constituents. Among the tested fractions, the ethyl acetate extract contained the highest measured concentrations of phenolic acids and flavonoids and showed the lowest numerical DPPH IC_50_ value. LC–MS/MS and GC–MS analyses indicated a chemically diverse profile comprising tentatively annotated amino acids, organic acids, nucleosides and related compounds, flavonoids, fatty acid derivatives, hydrocarbons, and aldehydes. GC–MS analysis of the hydrolyzed polysaccharide-enriched fraction also provided a provisional profile of its sugar-related components. Lipid analysis revealed selected peaks tentatively assigned as fatty acid methyl esters and components of the unsaponifiable fraction, whereas the volatile profile contained several lipid-derived aldehydes and related compounds. Preliminary antimicrobial screening showed inhibitory activity against selected bacterial and fungal pathogens, suggesting the presence of constituents with antimicrobial potential. Together, these results highlight the nutritional and phytochemical value of the examined SF sample and are consistent with its traditional dietary use. They also provide a scientific basis for further evaluation of SF as a potential source of ingredients for food and related applications. Further research should focus on compound isolation and structural confirmation, comprehensive analytical validation, bioavailability and mechanistic studies, toxicological and safety assessment, validation in appropriate food models, and in vivo and clinical evaluation to further evaluate its potential for incorporation into functional-food and nutraceutical formulations.

## Figures and Tables

**Table 1:** Nutritional composition of the SF sample.

S.N.	Composition	Result (g/100 g)
1	Total Solid	96.57
2	Ash	3.35
3	Fat	5.95
4	Protein	22.69
5	Fiber	1.20
6	Carbohydrate	63.38

**Table 2:** Total phenolics (TPC) and TFC of SF extracts.

Sample of SF Extract	TPC (mg GAE/g Extract)	TFC (mg RE/g Extract)
Hexane	7.20 ± 0.79	3.60 ± 0.14
Ethyl Acetate	33.61 ± 0.20	21.90 ± 0.07
Butanol	19.56 ± 1.09	5.11 ± 0.45
Aqueous	5.75 ± 0.54	0.00 ± 0.00

Values are expressed as mean ± SD of three repeated measurements of each sample.

**Table 3:** Elemental Composition of the SF Sample Determined by ICP Analysis (mg/kg).

Category	Element	Concentration (mg/kg)
Macro elements	P	3000
	K	1700
	Mg	975
	Na	700
	Ca	300
Trace Elements	Mn	75
	B	64
	Fe	48
	Zn	15
	Cu	10
	Al	6.25
	Ni	1.25
	Se	0.05
	Co	0.05
	Cr	0.05
Toxic Elements	Pb	<1
	Cd	<0.05
	As	<0.05
	Hg	<0.05

**Table 4:** Calibration parameters for the quantitative LC–MS/MS analysis of phenolic compounds in Samh flour extracts.

Compound	Regression Equation	R^2^	RSD (%)	LOD (µg/mL)	LOQ (µg/mL)
**Gallic acid**	y = 4.11883 × 10^6^ x − 6.18063 × 10^4^	0.993290	9.86	0.1155	0.3501
**Caffeic acid**	y = 2.41201 × 10^7^ x − 1.18433 × 10^5^	0.995454	6.61	0.0797	0.2415
**Rutin**	y = 1.07383 × 10^7^ x − 5.72083 × 10^4^	0.995533	9.31	0.0936	0.2836
**Coumaric acid**	y = 6.09201 × 10^7^ x − 1.34829 × 10^4^	0.995123	6.46	0.0660	0.2001
**Vanillin**	y = 2.06145 × 10^5^ x − 1.72615 × 10^3^	0.996407	4.62	0.0552	0.1672
**Naringenin**	y = 5.87360 × 10^4^ x − 3.05530 × 10^2^	0.994857	5.72	0.0689	0.2087
**Quercetin**	y = 3.38549 × 10^7^ x − 2.04308 × 10^5^	0.993531	7.04	0.0707	0.2141
**Ellagic acid**	y = 1.46451 × 10^6^ x + 1.46592 × 10^4^	0.993553	5.28	0.0662	0.2006
**3,4-Dihydroxybenzoic acid**	y = 4.58302 × 10^6^ x − 1.83190 × 10^4^	0.995912	4.67	0.0564	0.1708
**Hesperetin**	y = 1.17686 × 10^7^ x − 2.49228 × 10^4^	0.997195	4.88	0.0496	0.1503
**Cinnamic acid**	y = 7.94963 × 10^4^ x + 1.29917 × 10^3^	0.996897	3.93	0.0501	0.1518
**Methyl gallate**	y = 9.40942 × 10^7^ x + 6.23545 × 10^4^	0.993305	8.73	0.0895	0.2711
**Kaempferol**	y = 2.02140 × 10^6^ x − 3.97050 × 10^3^	0.995101	7.04	0.0716	0.2169
**Ferulic acid**	y = 3.53860 × 10^6^ x + 9.59489 × 10^2^	0.993689	9.44	0.0967	0.2930
**Syringic acid**	y = 1.41107 × 10^6^ x + 1.17610 × 10^3^	0.995167	6.19	0.0635	0.1925
**Apigenin**	y = 1.72619 × 10^5^ x + 3.23914 × 10^3^	0.995054	5.06	0.0649	0.1966
**Catechin**	y = 1.11140 × 10^6^ x + 1.53978 × 10^3^	0.992572	8.41	0.0864	0.2618
**Daidzein**	y = 1.01614 × 10^7^ x + 3.26134 × 10^4^	0.985864	14.39	0.1487	0.4506
**Chlorogenic acid**	y = 1.84148 × 10^7^ x − 4.59551 × 10^4^	0.995759	6.02	0.0611	0.1851
**Resveratrol**	y = 3.27587 × 10^6^ x + 2.29656 × 10^4^	0.981966	8.58	0.0898	0.2721
**Rosmarinic acid**	y = 3.04549 × 10^7^ x − 8.67007 × 10^4^	0.995384	6.42	0.0651	0.1971

**Table 5:** Quantitative LC–MS/MS profiling of selected phenolic and related compounds in different solvent fractions of SF extract (µg/g).

Component Name	Butanol (µg/g)	Ethyl Acetate (µg/g)	Water (µg/g)
Gallic acid (168.9/124.9)	ND	12.3589	ND
Caffeic acid (178/135)	ND	80.625	ND
Rutin (609/299.9)	1.2257	12.1071	7.3661
Coumaric acid (162.9/119)	1.1698	102.0893	0.3071
Vanillin (151/136)	55.7963	77.6607	ND
Naringenin (271/151)	305.3704	248.3929	ND
Quercetin (301/151)	6.1833	67.9107	1.1195
Ellagic acid (301/145)	ND	ND	ND
3,4-Dihydroxybenzoic acid (152.9/109)	30.2037	2732.1429	35.5
Hesperetin (301/136)	ND	ND	ND
Cinnamic acid (146.9/77)	51.7037	111.6607	ND
Methyl gallate (183/124)	ND	1.4043	ND
Kaempferol (284.7/93)	25.7778	225.5357	ND
Ferulic acid (192.8/133.9)	5.8833	410.3571	0.8815
Syringic acid (196.8/181.9)	1.0272	69.0179	ND
Apigenin (269/151)	ND	56.5357	ND
Catechin (288.8/109)	ND	ND	ND
Daidzein (253/132)	ND	ND	ND
Chlorogenic acid (353/191)	0.7976	2.4661	2.9290
Resveratrol (227/185)	ND	ND	ND
Rosmarinic acid (359.1/161)	0.6067	0.7669	ND

ND: Non detectable.

**Table 6:** LC–MS–Based Metabolite Identification in the Ethyl Acetate Extract.

ID	RT (min)	*m*/*z*	Type	Metabolite Name
146	1.163067	148.9495	[M+H]+	w/o MS2: trans-Cinnamate acid
158	1.2717	108.9377	[M+H]+	w/o MS2: 1,4-Benzoquinone
172	1.2717	244.9642	[M+H]+	w/o MS2: alpha-L-Fucose 1-phosphate
206	1.372133	364.9464	[M+H]+	w/o MS2: Xanthosine-5′-monophosphate
227	1.463633	118.056	[M+H]+	w/o MS2: Indole
256	1.565967	104.0395	[M+H]+	N, N-Dimethylglycine
260	1.565967	128.0722	[M+H]+	w/o MS2:Isoguvacine
275	1.652367	109.9562	[M+H]+	w/o MS2: Hypotaurine
361	1.87595	133.1077	[M+H]+	w/o MS2:L-Asparagine
444	2.418017	147.0691	[M+H]+	w/o MS2: L-Glutamine
822	4.666017	172.9538	[M+H]+	w/o MS2: Glycerol-2-phosphate
889	5.157583	160.9672	[M+H]+	w/o MS2: L-beta-homoglutamine
921	5.44515	174.9689	[M+H]+	w/o MS2: cis-Aconitate acid
943	5.751283	162.9497	[M+H]+	w/o MS2:3-Hydroxy-3-methylglutaric acid
954	5.751283	324.0748	[M+H]+	w/o MS2: Cytidine-5′-monophosphate
983	5.951217	165.0828	[M+H]+	w/o MS2: p-Coumaric acid
1094	6.589983	399.1041	[M+H]+	w/o MS2: S-Adenosyl-L-methionine
1148	6.948383	457.2188	[M+H]+	w/o MS2: Riboflavin-5′-monophosphate
1174	7.130533	211.0916	[M+H]+	w/o MS2: Sinapyl alcohol
1197	7.23005	190.1137	[M+H]+	w/o MS2: N-Acetylglutamate
1280	7.607467	299.1877	[M+H]+	w/o MS2: Methyl octadecanoate
1285	7.607467	597.1953	[M+H]+	w/o MS2: Eriodictyol-7-O-neohesperidoside
1462	8.262234	349.1144	[M+H]+	w/o MS2: Inosine-5′-monophosphate
1468	8.262234	625.1606	[M+H]+	w/o MS2: Isorhamnetin-3-O-rutinoside
1517	8.4403	339.1804	[M+H]+	w/o MS2: 5-Aminoimidazole-4-carboxamide ribonucleotide
1556	8.607284	159.0343	[M+H]+	w/o MS2: Allantoin
1678	8.973783	184.0767	[M+H]+	w/o MS2:4-Pyridoxate
1770	9.15015	343.2202	[M+H]+	w/o MS2:D-(+)-Maltose
2164	9.976183	243.1	[M+H]+	w/o MS2: Thymidine
2190	10.0573	150.0692	[M+H]+	w/o MS2:L-Methionine
2356	10.46697	332.9657	[M+H]+	w/o MS2:2′-Deoxyinosine-5′-monophosphate
2382	10.56273	137.1755	[M+H]+	w/o MS2: Sabinene
2494	10.89492	204.0721	[M+H]+	w/o MS2: Isopentenyladenine
2706	11.43225	191.9603	[M+H]+	w/o MS2:5-Hydroxyindoleacetic acid
2730	11.51228	300.9508	[M+H]+	w/o MS2: Kaempferide
2833	11.7579	316.9241	[M+H]+	w/o MS2:3′-Methoxy-4′,5,7-trihydroxyflavonol
2925	11.89783	355.1118	[M+H]+	w/o MS2: Chlorogenic acid
3439	13.20943	285.1004	[M+H]+	w/o MS2: Xanthosine
3506	13.3808	228.0968	[M+H]+	w/o MS2: 2′-Deoxycytidine
3920	14.39062	387.0887	[M+H]+	w/o MS2:1-O-β-D-glucopyranosyl sinapate
4850	15.54462	429.0106	[M+H]+	w/o MS2: Inosine-5′-diphosphate
5204	16.55242	335.1748	[M+H]+	w/o MS2: β-Nicotinamide mononucleotide
5997	18.50193	244.1767	[M+H]+	w/o MS2: Cytidine
6034	18.5695	319.0619	[M+H]+	w/o MS2: Myricetin
6289	19.1448	268.0894	[M+H]+	w/o MS2: Adenosine
6404	19.38662	277.075	[M+H]+	w/o MS2: L-Saccharopine
6583	19.77038	361.2072	[M+H]+	w/o MS2: Rosmarinic acid
7118	20.85102	297.2858	[M+H]+	w/o MS2: Phytol
7510	21.72945	245.1342	[M+H]+	w/o MS2: Uridine
7513	21.72945	263.1338	[M+H]+	w/o MS2: Sorbitol-6-phosphate
9029	23.60153	265.1975	[M+H]+	w/o MS2:cis/trans-Abscisic acid
10416	24.71632	328.2766	[M+H]+	w/o MS2: Scoulerine
12000	25.70427	284.0774	[M+H]+	w/o MS2: Guanosine
13510	27.02432	118.0611	[M+H]+	w/o MS2:5-Aminovaleric acid
15232	28.19573	336.1621	[M+H]+	w/o MS2: N-6-Isopentenyladenosine

RT represents retention time; *m*/*z* denotes the mass-to-charge ratio; and [M+H]+ indicates protonated molecular ions detected in positive ionization mode. w/o MS2 denotes metabolite annotations based on precursor-ion (MS1) data obtained during the general LC–MS screening.

**Table 7:** LC–MS–Based Metabolite Identification in the Butanol SF Extract.

ID	RT (min)	*m*/*z*	Type	Metabolite Name
263	1.3894	104.0512	[M+H]+	w/o MS2: N-Methylalanine
274	1.3894	365.034	[M+H]+	w/o MS2: Xanthosine-5′-monophosphate
304	1.443	123.1772	[M+H]+	w/o MS2: Nicotinamide
306	1.443	136.0438	[M+H]+	w/o MS2: S-Methyl-L-cysteine
361	1.629317	104.1299	[M+H]+	w/o MS2: N, N-Dimethylglycine
374	1.703083	124.0641	[M+H]+	w/o MS2: Nicotinic acid
404	1.847667	179.0799	[M+H]+	w/o MS2: 4-Methoxycinnamic acid
413	1.901867	118.1766	[M+H]+	w/o MS2: Glycine betaine
456	2.020267	144.0868	[M+H]+	w/o MS2: 4-Methyl-5-thiazoleethanol
490	2.209133	185.0779	[M+H]+	w/o MS2: 3,4-Dihydroxymandelic acid
643	2.982067	195.096	[M+H]+	w/o MS2: Ferulic acid
1096	6.019967	192.9768	[M+H]+	w/o MS2: Scopoletin
1129	6.2271	262.9247	[M+H]+	w/o MS2: Sorbitol-6-phosphate
1175	6.467017	169.0685	[M+H]+	w/o MS2: Pyridoxamine
1230	6.845083	211.0789	[M+H]+	w/o MS2: Sinapyl alcohol
1262	6.955534	187.0895	[M+H]+	w/o MS2: 3-Phospho-D-glycerate
1287	7.0753	317.0232	[M+H]+	w/o MS2: 3′-Methoxy-4′,5,7-trihydroxyflavonol
1349	7.316283	137.1785	[M+H]+	w/o MS2: Hypoxanthine
1363	7.316283	166.2427	[M+H]+	w/o MS2: L-Phenylalanine
1370	7.316283	183.1761	[M+H]+	w/o MS2: Syringaldehyde

RT represents retention time; *m*/*z* denotes the mass-to-charge ratio; [M+H]+ indicates protonated molecular ions detected in positive ionization mode. w/o MS2 denotes metabolite annotations based on precursor-ion (MS1) data obtained during the general LC–MS screening.

**Table 8:** LC–MS–Based Metabolite Identification in the Aqueous SF Extract.

ID	RT (min)	*m*/*z*	Type	Metabolite Name
29	0.5566	162.9345	[M+H]+	w/o MS2: 3-Hydroxy-3-Methylglutaric acid
76	1.037667	137.0364	[M+H]+	w/o MS2: Hypoxanthine
138	1.316967	364.946	[M+H]+	w/o MS2: Xanthosine-5′-monophosphate
282	1.384867	245.0671	[M+H]+	w/o MS2: Uridine
563	1.441917	109.929	[M+H]+	w/o MS2: Hypotaurine
566	1.441917	112.1376	[M+H]+	w/o MS2: Histamine
1218	1.75765	345.0113	[M+H]+	w/o MS2: Maltitol
1847	2.248917	126.0274	[M+H]+	w/o MS2: 5-Methylcytosine
2037	2.4938	103.9789	[M+H]+	w/o MS2: N, N-Dimethylglycine
2212	3.065767	185.0348	[M+H]+	w/o MS2: 3,4-Dihydroxymandelate
2458	4.755567	126.029	[M+H]+	w/o MS2: 5-Methylcytosine
2534	5.58595	118.1401	[M+H]+	w/o MS2: 5-Aminovaleric acid
2536	5.58595	137.1398	[M+H]+	w/o MS2:Sabinene
2643	6.005667	177.0232	[M+H]+	w/o MS2: N-Carbamoyl-L-Aspartic acid
2913	6.896567	399.2268	[M+H]+	w/o MS2: S-Adenosyl-L-methionine
3202	7.473217	251.0753	[M+H]+	w/o MS2:gamma-Glu-Cys
3220	7.473217	373.2508	[M+H]+	w/o MS2: Eleutheroside B
3327	7.626367	597.2858	[M+H]+	w/o MS2: Eriodictyol-7-O-neohesperidoside
3418	7.8573	227.1872	[M+H]+	w/o MS2: Methyl dihydrojasmonate
3656	8.269934	198.0501	[M+H]+	w/o MS2: 3,4-Dihydroxy-L-phenylalanine
3657	8.269934	215.1163	[M+H]+	w/o MS2: Harmaline
3666	8.269934	317.2106	[M+H]+	w/o MS2: 3′-Methoxy-4′,5,7-trihydroxyflavonol
3736	8.42385	127.0589	[M+H]+	w/o MS2: Thymine
4444	9.43465	137.0358	[M+H]+	w/o MS2: Hypoxanthine
4646	9.8598	243.0401	[M+H]+	w/o MS2: Thymidine
5272	11.7289	123.019	[M+H]+	w/o MS2: Nicotinamide
5303	11.82847	317.0315	[M+H]+	w/o MS2: 3′-Methoxy-4′,5,7-trihydroxyflavonol
5580	12.83907	231.026	[M+H]+	w/o MS2: D-Ribose 5-phosphate
5696	13.37072	228.0957	[M+H]+	w/o MS2: 2′-Deoxycytidine
5702	13.37072	285.056	[M+H]+	w/o MS2: Acacetin
5724	13.48898	158.1446	[M+H]+	w/o MS2: N-Tigloylglycine
6112	14.41218	387.0219	[M+H]+	w/o MS2: 1-O-β-D-glucopyranosyl sinapate
7170	17.6063	241.0411	[M+H]+	w/o MS2: L-Cystine
7296	18.24668	581.1214	[M]+	w/o MS2: Cyanidin-3-O-(xyl-glucoside)
7510	19.11032	268.0457	[M+H]+	w/o MS2: Adenosine
7704	19.85903	148.9666	[M+H]+	w/o MS2: trans-Cinnamate acid
9961	26.05425	284.1525	[M+H]+	w/o MS2: Guanosine
11759	28.41182	144.1761	[M+H]+	w/o MS2: 4-Methyl-5-thiazoleethanol

RT represents retention time; *m*/*z* denotes the mass-to-charge ratio; [M+H]+ indicates protonated molecular ions detected in positive ionization mode. w/o MS2 denotes metabolite annotations based on precursor-ion (MS1) data obtained during the general LC–MS screening.

**Table 9:** GC–MS Profile of Selected Trimethylsilylated Components Detected in the Hydrolysate of the Polysaccharide-Enriched SF Fraction.

RT (min)	Area %	Compound Name	SI	Molecular Weight	Molecular Formula
4.52	0.01	Pyridine ^a^	800	79	C_5_H_5_N
5.34	0.01	2,4-Pentadienenitrile	781	79	C_5_H_5_N
8.28	0.07	Trimethylsilyl (2E)-2-hexenoate	762	186	C_9_H_18_O_2_Si
8.77	0.16	Pentasiloxane, dodecamethyl- ^b^	951	384	C_12_H_36_O_4_Si_5_
9.53	0.08	Trimethylsilyl ether of glycerol	839	308	C_12_H_32_O_3_Si_3_
10.67	0.15	Phenol, 2,6-dimethoxy-	886	154	C_8_H_10_O_3_
12.18	0.13	Phenol, 4,5-dimethoxy-2-methyl-	877	168	C_9_H_12_O_3_
14.98	0.55	D-Ribose, 2,3,4,5-tetrakis-O-(trimethylsilyl)-	867	438	C_17_H_42_O_5_Si_4_
15.30	0.42	α-DL-Arabinofuranoside, methyl 2,3,5-tris-O-(trimethylsilyl)-	793	380	C_15_H_36_O_5_Si_3_
15.50	0.46	D-Ribopyranose, 1,2,3,4-tetrakis-O-(trimethylsilyl)-	809	438	C_17_H_42_O_5_Si_4_
18.29	13.28	Glucopyranose, 1,2,3,4,6-pentakis-O-(trimethylsilyl)-, D-	866	540	C_21_H_52_O_6_Si_5_
18.82	11.03	Hexopyranose, 1,2,3,4,6-pentakis-O-(trimethylsilyl)-	929	540	C_21_H_52_O_6_Si_5_
19.17	9.10	Mannose, 6-deoxy-2,3,4,5-tetrakis-O-(trimethylsilyl)-, L-	882	452	C_18_H_44_O_5_Si_4_
19.90	9.66	Talose, 2,3,4,5,6-pentakis-O-(trimethylsilyl)-	935	540	C_21_H_52_O_6_Si_5_
21.23	0.41	Trimethyl(oxy)silane	915	612	C_24_H_60_O_6_Si_6_
25.64	0.18	1,2-Benzenedicarboxylic acid, diisooctyl ester ^c^	902	390	C_24_H_38_O_4_
28.98	0.71	Melibiose, octakis(trimethylsilyl)-	912	918	C_36_H_86_O_11_Si_8_

RT represents retention time, Area (%) indicates relative abundance, and SI refers to the similarity index obtained from library matching. Identified compounds are reported as trimethylsilyl (TMS) derivatives based on GC–MS analysis. ^a^ Derivatization reagent-related compound; ^b^ probable siloxane background contaminant; ^c^ probable plasticizer contaminant. These compounds were excluded from the biological interpretation. Trimethylsilylated sugar derivatives represent expected products of the derivatization process.

**Table 10:** GC–MS Profile of Selected FAME-Related Peaks in the Methylated Saponifiable Fraction of SF Fixed Oil.

RT (min)	Area %	Compound Name	SI	Molecular Weight	Molecular Formula
27.60	0.16	Methyl tetradecanoate	929	242	C_15_H_30_O_2_
29.36	0.18	Pentadecanoic acid, methyl ester	903	256	C_16_H_32_O_2_
30.71	0.28	9-Hexadecenoic acid, methyl ester (Z)	916	268	C_17_H_32_O_2_
31.07	5.73	Hexadecanoic acid, methyl ester (methyl palmitate)	659	270	C_17_H_34_O_2_
32.20	0.58	Hexadecanoic acid, 14-methyl-, methyl ester	861	284	C_18_H_36_O_2_
32.65	0.17	Heptadecanoic acid, methyl ester	931	284	C_18_H_36_O_2_
33.24	0.21	Octadecenoic acid, methyl ester isomer	820	296	C_19_H_36_O_2_
33.77	19.81	9,12-Octadecadienoic acid (Z,Z)-, methyl ester (methyl linoleate)	790	294	C_19_H_34_O_2_
34.19	1.39	Octadecanoic acid, methyl ester	941	298	C_19_H_38_O_2_
34.58	0.32	9,12-Octadecadienoic acid (Z, Z), methyl ester	923	294	C_19_H_34_O_2_
34.87	0.21	Cyclopropaneoctanoic acid, 2-octyl-, methyl ester	830	310	C_20_H_38_O_2_
35.25	0.65	Octadecanoic acid, 17-methyl-, methyl ester	850	312	C_20_H_40_O_2_
36.54	0.82	Eicosanoic acid, methyl ester	816	326	C_21_H_42_O_2_
36.71	0.81	11-Eicosenoic acid, methyl ester	909	324	C_21_H_40_O_2_
37.06	0.97	Eicosanoic acid, methyl ester	925	326	C_21_H_42_O_2_
38.04	0.23	Heneicosanoic acid, methyl ester	816	340	C_22_H_44_O_2_

RT indicates retention time; Area (%) represents relative abundance; and SI refers to the similarity index obtained from GC–MS library matching. Identified compounds are reported as fatty acid methyl esters (FAMEs) formed after saponification.

**Table 11:** GC–MS Profile of Selected Components in the Unsaponifiable Fraction of SF Fixed Oil.

RT (min)	Area %	Compound Name	SI	Molecular Weight	Molecular Formula
12.74	0.27	TETRADECANE	807	198	C_14_H_30_
35.24	1.04	DOCOSANE	860	310	C_22_H_46_
36.65	0.95	DOCOSANE	855	310	C_22_H_46_
38.01	0.31	DOCOSANE	847	310	C_22_H_46_
51.59	14.27	9-Hexadecenoicacid,eicosyl ester, (Z)-	635	534	C_36_H_70_O_2_

RT indicates retention time; Area (%) represents relative abundance; and SI refers to the similarity index obtained from GC–MS library matching. Compounds were identified in the unsaponifiable fraction by GC–MS analysis.

**Table 12:** GC–MS Profile of Volatile Compounds.

RT (min)	Area %	Compound Name	SI	Molecular Weight	Molecular Formula
7.19	0.50	Furan, 2-pentyl-	904	138	C_9_H_14_O
7.42	0.13	Decane	826	142	C_10_H_22_
9.00	0.54	2,3,3-Trimethyloctane	853	156	C_11_H_24_
9.16	0.22	Undecane	806	156	C_11_H_24_
10.18	0.18	Undecane	902	156	C_11_H_24_
11.85	0.67	2-Nonenal, (E)-	883	140	C_9_H_16_O
14.70	2.84	2-Decenal, (E)-	918	154	C_10_H_18_O
15.22	0.49	Hexadecane	872	226	C_16_H_34_
15.59	9.55	2,4-Decadienal, (E, E)-	891	152	C_10_H_16_O
16.24	37.00	(5Z)-3-Methyl-1,5-heptadiene	585	110	C_8_H_14_
16.46	0.64	Dodecane, 2,6,11-trimethyl-	855	212	C_15_H_32_
16.70	0.26	Pentadecane	827	212	C_15_H_32_
17.11	0.80	3-Cyclohexene-1-methanol, α,α,4-trimethyl-, acetate	899	196	C_12_H_20_O_2_
17.25	0.53	Naphthalene, 1,2-dihydro-1,1,6-trimethyl-	906	172	C_13_H_16_
17.45	3.98	2-Undecenal	869	168	C_11_H_20_O
18.16	0.51	1-Tetradecene	828	196	C_14_H_28_
18.37	0.91	Tetradecane	950	198	C_14_H_30_
18.60	0.24	Dodecanal	817	184	C_12_H_24_O
19.72	0.44	6,10-Dimethyl-5,9-undecadien-2-one	833	194	C_13_H_22_O
20.23	1.11	1-Dodecanol	891	186	C_12_H_26_O
20.78	0.56	Hexadecane	856	226	C_16_H_34_
21.86	0.49	Heptadecane, 2,6,10,15-tetramethyl-	828	296	C_21_H_44_
23.22	1.97	Nonadecane	877	268	C_19_H_40_
25.12	0.34	Benzene, 1,4-dimethyl-2-[(4-methylphenyl)methyl]-	856	210	C_16_H_18_
26.64	0.36	Hexadecane, 2,6,10,14-tetramethyl-	826	282	C_20_H_42_
26.99	0.40	Heptadecane, 3-methyl-	833	254	C_18_H_38_
28.14	0.38	Isopropyl myristate	807	270	C_17_H_34_O_2_
28.54	0.67	2-Pentadecanone, 6,10,14-trimethyl-	838	268	C_18_H_36_O

RT indicates retention time; Area (%) represents relative abundance; and SI refers to the similarity index obtained from GC–MS library matching.

**Table 13:** Inhibition-zone diameters (mm) of SF extracts tested at 50 mg/mL using 75 µL per well (3.75 mg/well).

Test Organism	Ethyl Acetate		Butanol		Aqueous		Reference
	(A)	(B)	(A)	(B)	(A)	(B)	
*Escherichia coli*	21.0	22.0	15.0	15.0	Nil	Nil	15.0 (CN)
*Pseudomonas aeruginosa*	13.0	13.0	17.0	17.0	Nil	Nil	13.0 (CN)
*Bacillus subtilis*	12.0	12.0	14.0	14.0	Nil	Nil	15.0 (CN)
*Bacillus cereus*	12.0	11.0	14.0	13.0	Nil	Nil	19.0 (CN)
*Staphylococcus aureus*	12.0	12.0	17.0	15.0	Nil	Nil	17.0 (CN)
*Candida albicans*	12.0	12.0	17.0	17.0	Nil	Nil	20.0 (MCZ)
*Aspergillus niger*	13.0	13.0	16.0	16.0	10.0	10.0	14.0 (MCZ)
*Aspergillus flavus*	15.0	15.0	22.0	22.0	Nil	Nil	12.0 (MCZ)

NOTE: A and B represent duplicate technical determinations of the inhibition-zone diameter for each extract against each test organism. Reference: CN, gentamicin 10 µg (standard antibiotic disc); MCZ, miconazole 50 µg (standard antifungal disc); NiL, no antimicrobial activity recorded.

**Table 14:** Minimum inhibitory concentrations of the active SF fractions and reference antimicrobial against the tested microorganisms.

Test Organism	Ethyl Acetate (mg/mL)	Butanol (mg/mL)	Reference Antimicrobial (mg/mL)	Reference (CN) (mg/mL)
*Escherichia coli*	5.0	7.5	Gentamicin (CN)	0.094
*Pseudomonas aeruginosa*	7.5	3.75	Gentamicin (CN)	0.125
*Bacillus subtilis*	10.0	7.5	Gentamicin (CN)	0.145
*Bacillus cereus*	10.0	7.5	Gentamicin (CN)	0.118
*Staphylococcus aureus*	7.5	5.0	Gentamicin (CN)	0.060
*Candida albicans*	7.5	3.5	ND	ND

CN, gentamicin; ND, not determined. The aqueous fraction was not subjected to MIC determination because it showed no or limited activity in the initial well-diffusion screening.

**Table 15:** Minimum bactericidal concentrations (MBCs) and minimum fungicidal concentration (MFC) of the active SF fractions.

Test Organism		Treated Sample	
		MBC (mg/mL)	
	Endpoint	Ethyl Acetate (mg/mL)	Butanol (mg/mL)
*Escherichia coli*	MBC	10	12.5
*Pseudomonas aeruginosa*	MBC	12.5	7.5
*Bacillus subtilis*	MBC	17.5	12.5
*Bacillus cereus*	MBC	17.5	12.5
*Staphylococcus aureus*	MBC	17.5	10.0
*Candida albicans*	MFC	12.5	7.5

MBC, minimum bactericidal concentration; MFC, minimum fungicidal concentration.

**Table 16:** MBC/MIC and MFC/MIC ratios of the active SF fractions.

Test Organism		Treated Sample	
		MBC	
	Ratio	Ethyl Acetate	Butanol
*Escherichia coli*	MBC/MIC	2.0	1.67
*Pseudomonas aeruginosa*	MBC/MIC	1.67	2.00
*Bacillus subtilis*	MBC/MIC	1.75	1.67
*Bacillus cereus*	MBC/MIC	1.75	1.67
*Staphylococcus aureus*	MBC/MIC	2.33	2.0
*Candida albicans*	MFC/MIC	1.6	2.14

All ratios are dimensionless. MBC, minimum bactericidal concentration; MFC, minimum fungicidal concentration; MIC, minimum inhibitory concentration.

## Data Availability

The original contributions presented in this study are included in the article/Supplementary Materials. Further inquiries can be directed to the corresponding author.

## Supplementary Materials

The following supporting information can be downloaded at: https://www.mdpi.com/article/10.3390/foods15213937/s1, Figures S1–S4. Agar well-diffusion assay photographs of Samh flour (SF) samples against the tested microorganisms, Figures S5–S13. Subculture plate photographs of Samh flour (SF) fractions against the tested microorganisms.; Table S1: DPPH radical-scavenging activity of SF fractions at different concentrations (µg/mL).
