# Supplementary material for: Compositional and Nutritional Analyses of Samh (Mesembryanthemum cryptanthum) Flour: Integrated Phenolic, Metabolomic, Fatty Acid, and Antimicrobial Activity Profiling
Source: Foods. 2026 Nov 3;15(21):3937. doi: 10.3390/foods15213937 (PMC13762468; doi:10.3390/foods15213937)
Supplement: Supplementary file 1 [file foods-15-03937-s001.zip › foods-4611170-supplementary.pdf]

Compositional and Nutritional Analyses of Samh (*Mesembryanthemum cryptanthum*) Flour: Integrated Phenolic, Metabolomic, Fatty Acid, and Antimicrobial Activity Profiling

Alanood A. Alfaleh [1], Tahreer M. Alraddadi [2], Maha A. Althaiban [1,*], Woroud A. Alsanei [1], Shatha H. Alaoufi [3] and Ebtihaj O. Alnasri [1,]

[1] Food and Nutrition Department, Faculty of Human Sciences and Design, King Abdulaziz University, P.O. Box 80200, Jeddah 21589, Saudi Arabia; aaalfaleh@kau.edu.sa (A.A.A.); walsanei@kau.edu.sa (W.A.A.); ealnassri@kau.edu.sa (E.O.A.)

[2] Chemistry Department, Al-Qunfudah University College, Umm Al-Qura University, P.O. Box 1109, Makkah 21912, Saudi Arabia; tmradade@uqu.edu.sa

[3] Department of Early Childhood, College of Education, Qassim University, P.O. Box 4518, Buraidah 52571, Saudi Arabia; shatha.alaoufi@qu.edu.sa

* Correspondence: mthaiban@kau.edu.sa

Supplementary Information (SI)

2.5. DPPH Assay

Table S1. DPPH radical-scavenging activity of SF fractions at different concentrations (µg/mL).

Extract	Concentration (µg/mL)	Scavenging (%)
Hexane Fraction.	10	8.34 ± 0.51
Hexane Fraction.	5	4.21 ± 0.34
Hexane Fraction.	2.5	−0.35 ± 0.03
Ethyl acetate Fraction.	10	75.07 ± 11.81
Ethyl acetate Fraction.	5	47.50 ± 6.50
Ethyl acetate Fraction.	2.5	26.69 ± 1.30
Butanol Fraction.	10	21.65 ± 1.22
Butanol Fraction.	5	2.02 ± 0.11
Butanol Fraction.	2.5	−3.46 ± 0.09
Water Fraction.	10	−6.15 ± 0.20
Water Fr.	5	−3.65 ± 0.11
Water Fr.	2.5	−6.93 ± 0.38

Antimicrobial activity by agar well diffusion

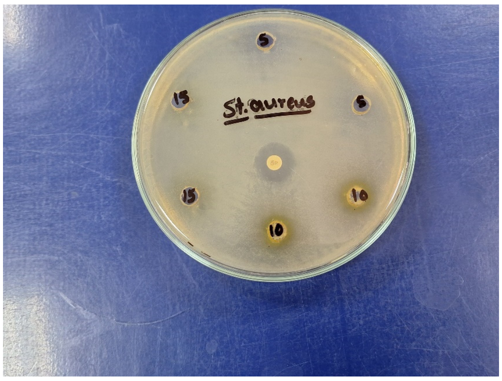

(A) Staphylococcus aureus

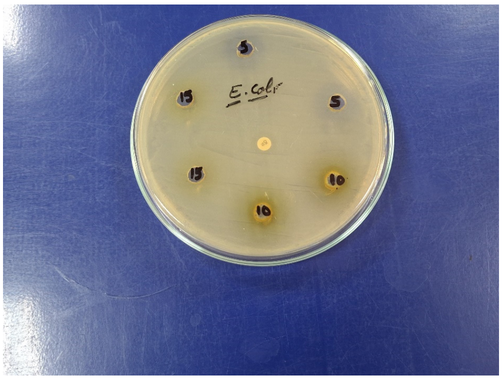

(B) Escherichia coli

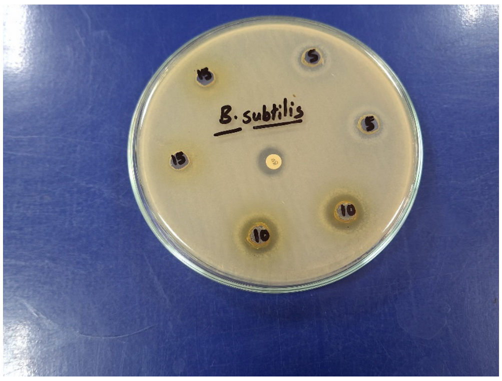

(C) Bacillus subtilis

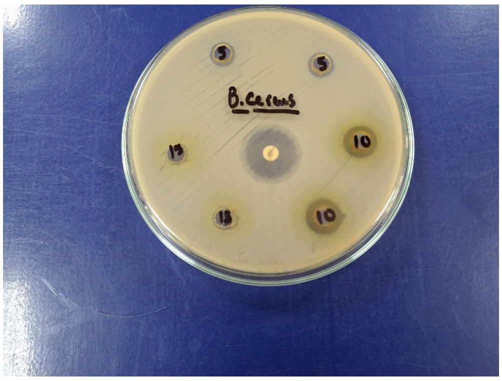

(D) Bacillus cereus

Figure S1. Agar well-diffusion photographs of SF samples against (A) Staphylococcus aureus; (B) Escherichia coli; (C) Bacillus subtilis; (D) Bacillus cereus. Plate codes: 5, ethyl acetate fraction; 10, butanol fraction; 15, aqueous fraction. Extracts: 50 mg/mL, 75 µL/well. Reference: gentamicin for bacteria and miconazole for fungi.

Antimicrobial activity by agar well diffusion

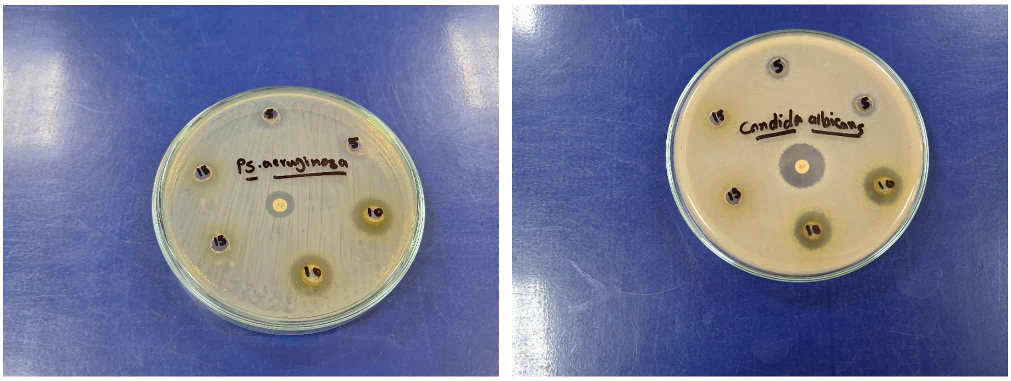

(A) Pseudomonas aeruginosa (B) Candida albicans

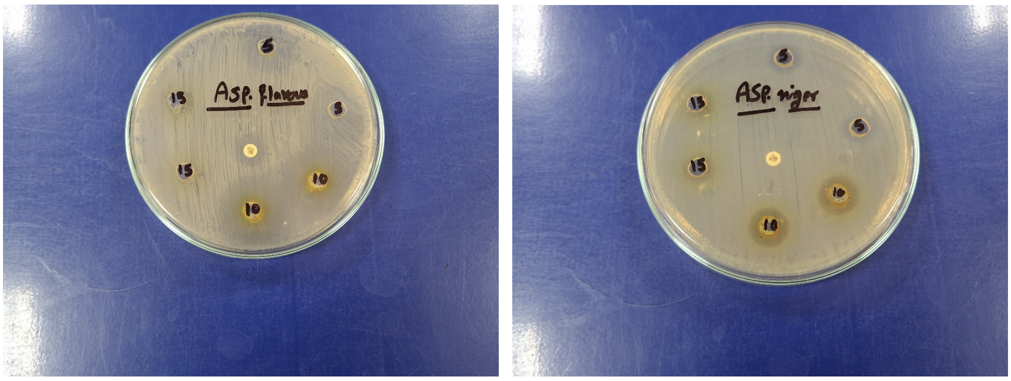

(C) Aspergillus flavus (D) Aspergillus niger

Figure S2. Agar well-diffusion photographs of SF samples against (A) Pseudomonas aeruginosa; (B) Candida albicans; (C) Aspergillus flavus; (D) Aspergillus niger. Plate codes: 5, ethyl acetate fraction; 10, butanol fraction; 15, aqueous fraction. Extracts: 50 mg/mL, 75 µL/well. Reference: gentamicin for bacteria and miconazole for fungi.

Antimicrobial activity by agar well diffusion

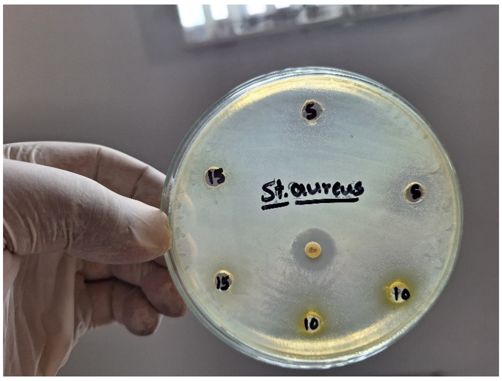

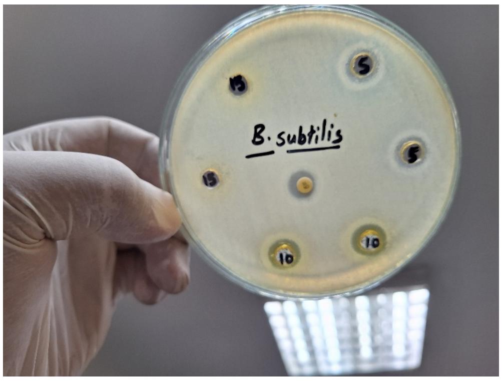

(A) Staphylococcus aureus (B) Bacillus subtilis

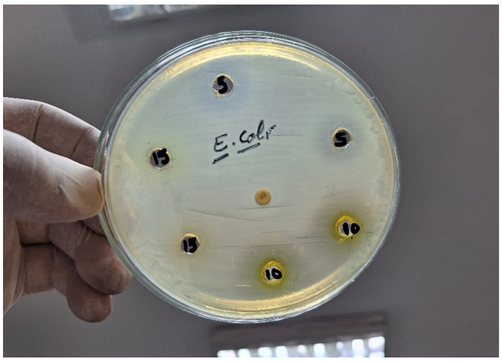

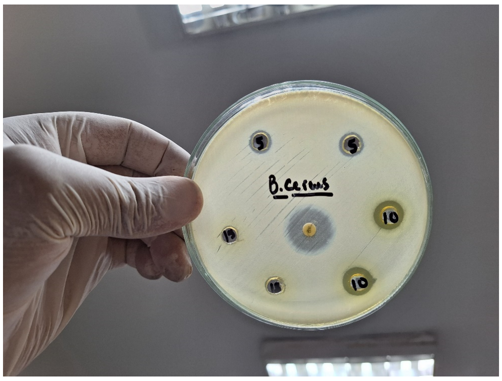

(C) Escherichia coli (D) Bacillus cereus

Figure S3. Agar well-diffusion photographs of SF samples against (A) Staphylococcus aureus; (B) Bacillus subtilis; (C) Escherichia coli; (D) Bacillus cereus. Plate codes: 5, ethyl acetate fraction; 10, butanol fraction; 15, aqueous fraction. Extracts: 50 mg/mL, 75 µL/well. Reference: gentamicin for bacteria and miconazole for fungi.

Antimicrobial activity by agar well diffusion

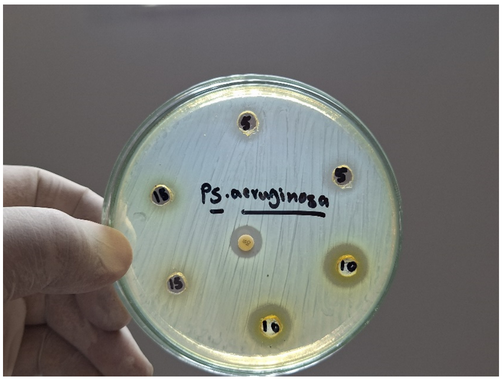

(A) Pseudomonas aeruginosa

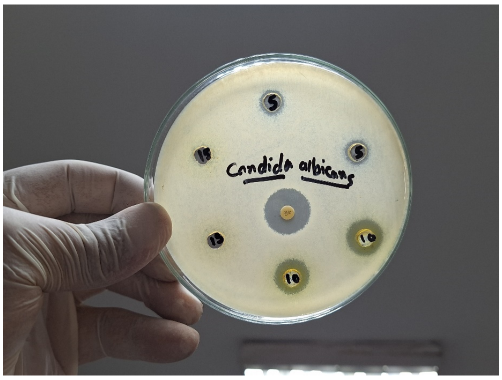

(B) Candida albicans

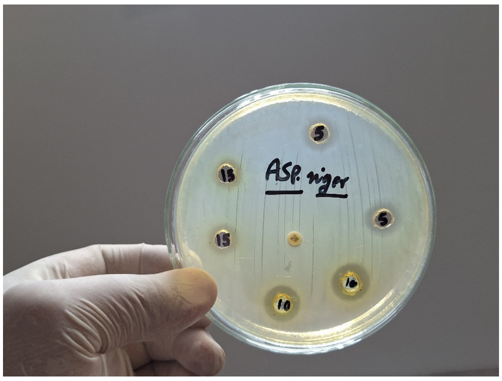

(C) Aspergillus niger

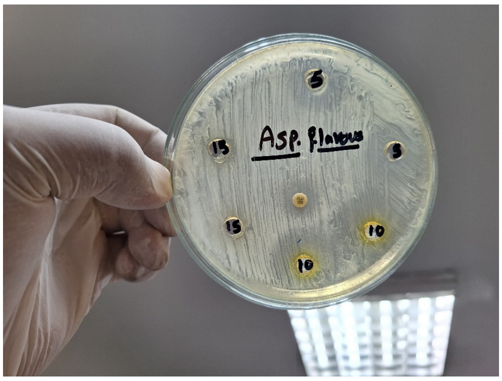

(D) Aspergillus flavus

Figure S4. Agar well-diffusion photographs of SF samples against (A) Pseudomonas aeruginosa; (B) Candida albicans; (C) Aspergillus niger; (D) Aspergillus flavus. Plate codes: 5, ethyl acetate fraction; 10, butanol fraction; 15, aqueous fraction. Extracts: 50 mg/mL, 75 µL/well. Reference: gentamicin for bacteria and miconazole for fungi.

Subculture plates for antimicrobial endpoint assessment

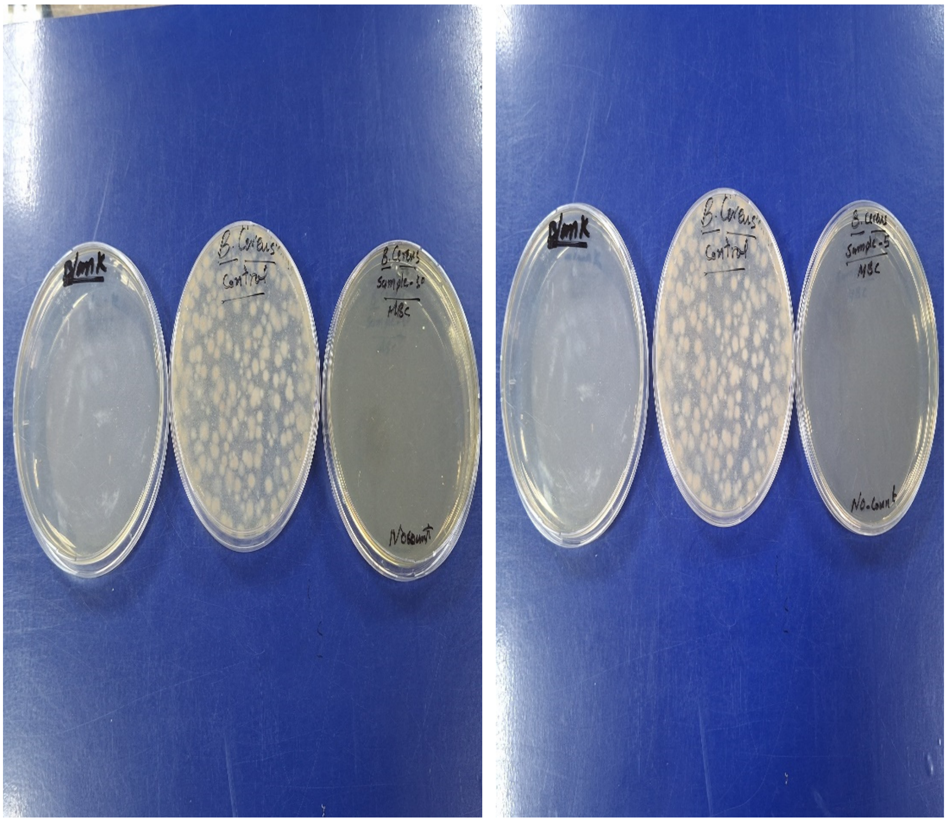

(A) Ethyl acetate fraction (B) Butanol fraction

Figure S5. Subculture photographs for Bacillus cereus: (A) the ethyl acetate fraction and (B) the butanol fraction. Plates, left to right: blank, organism control, and treated sample. Original codes: 5, ethyl acetate fraction; 10, butanol fraction.

Subculture plates for antimicrobial endpoint assessment

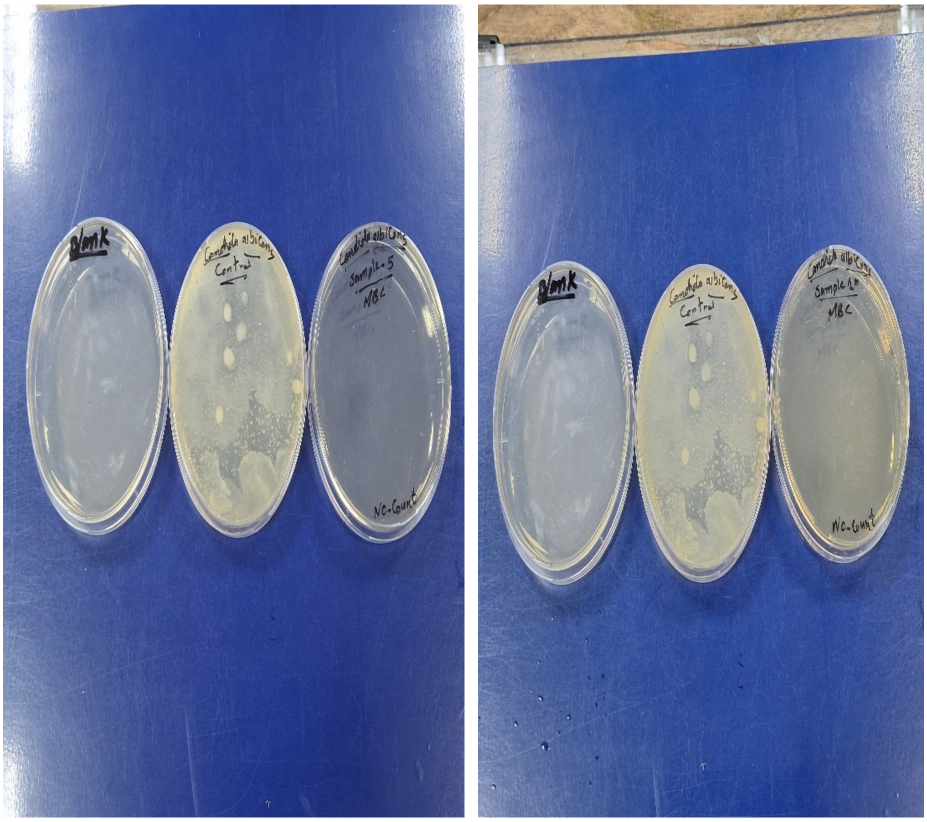

(A) Ethyl acetate fraction (B) Butanol fraction

Figure S6. Subculture photographs for Candida albicans: (A) the ethyl acetate fraction and (B) the butanol fraction. Plates, left to right: blank, organism control, and treated sample. Original codes: 5, ethyl acetate fraction; 10, butanol fraction.

Subculture plates for antimicrobial endpoint assessment

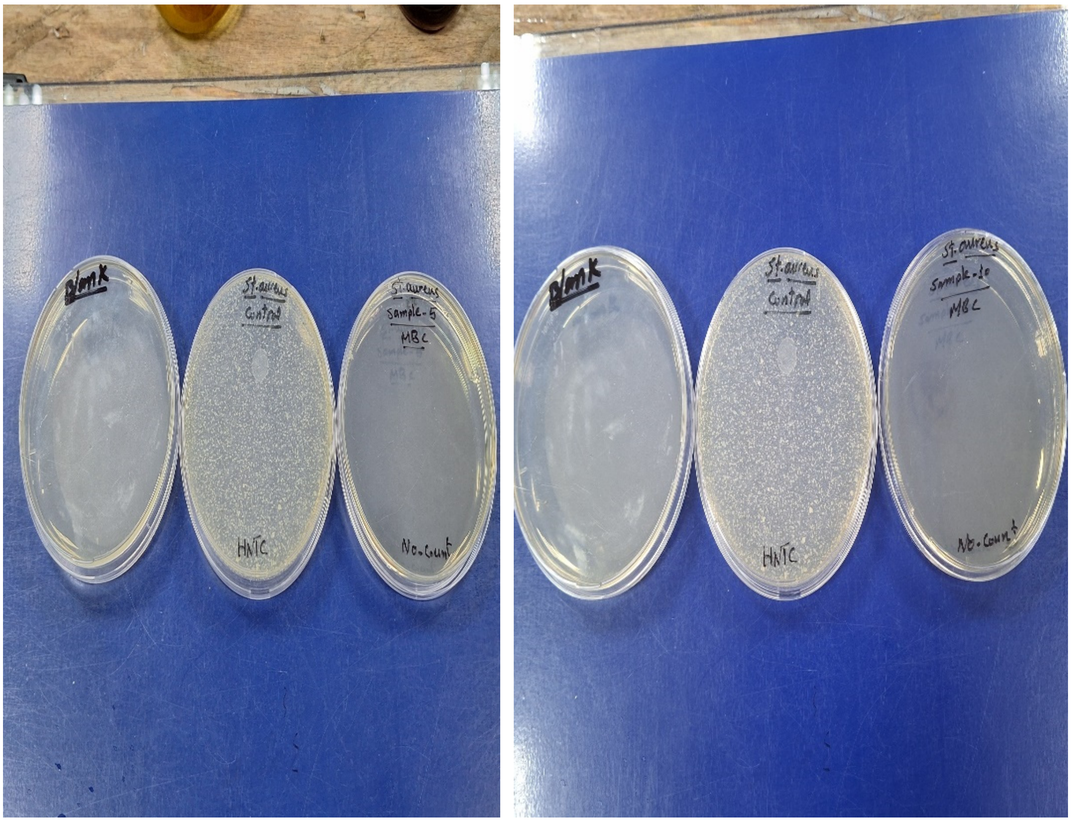

(A) Ethyl acetate fraction (B) Butanol fraction

Figure S7. Subculture photographs for Staphylococcus aureus: (A) the ethyl acetate fraction and (B) the butanol fraction. Plates, left to right: blank, organism control, and treated sample. Original codes: 5, ethyl acetate fraction; 10, butanol fraction.

Subculture plates for antimicrobial endpoint assessment

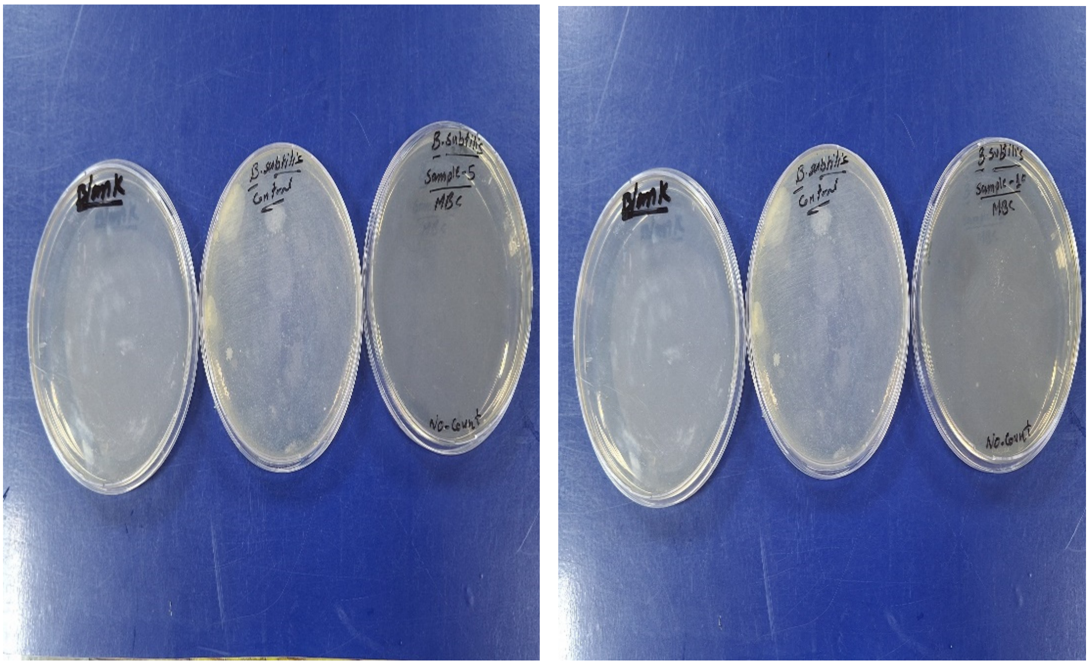

(A) Ethyl acetate fraction (B) Butanol fraction

Figure S8. Subculture photographs for Bacillus subtilis: (A) the ethyl acetate fraction and (B) the butanol fraction. Plates, left to right: blank, organism control, and treated sample. Original codes: 5, ethyl acetate fraction; 10, butanol fraction.

Subculture plates for antimicrobial endpoint assessment

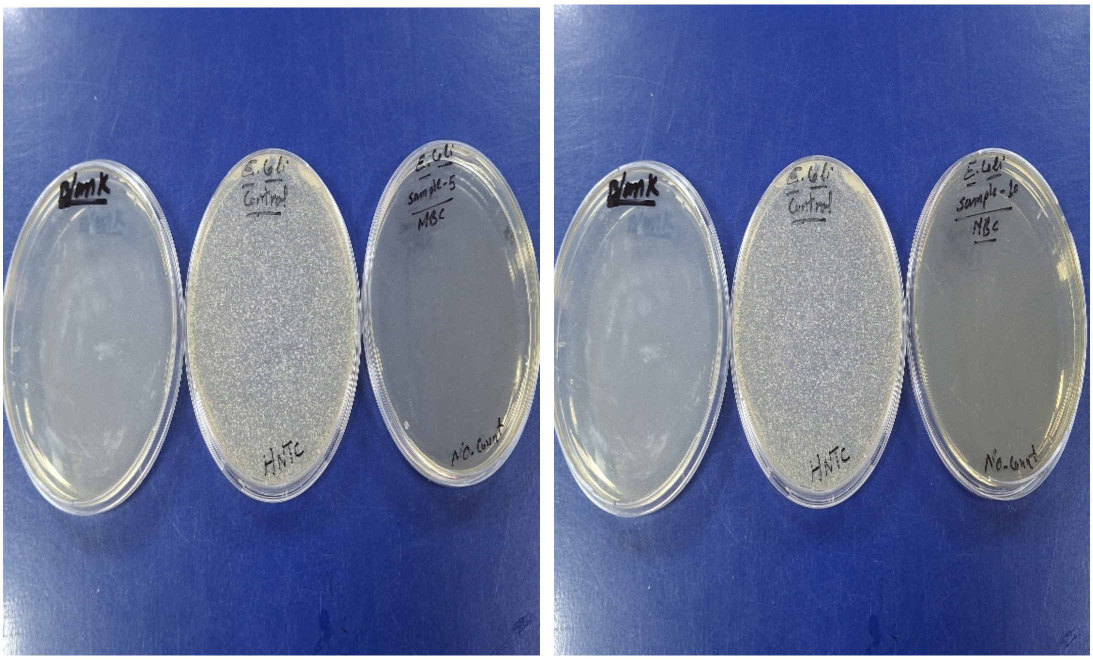

(A) Ethyl acetate fraction (B) Butanol fraction

Figure S9. Subculture photographs for Escherichia coli: (A) the ethyl acetate fraction and (B) the butanol fraction. Plates, left to right: blank, organism control, and treated sample. Original codes: 5, ethyl acetate fraction; 10, butanol fraction.

Subculture plates for antimicrobial endpoint assessment

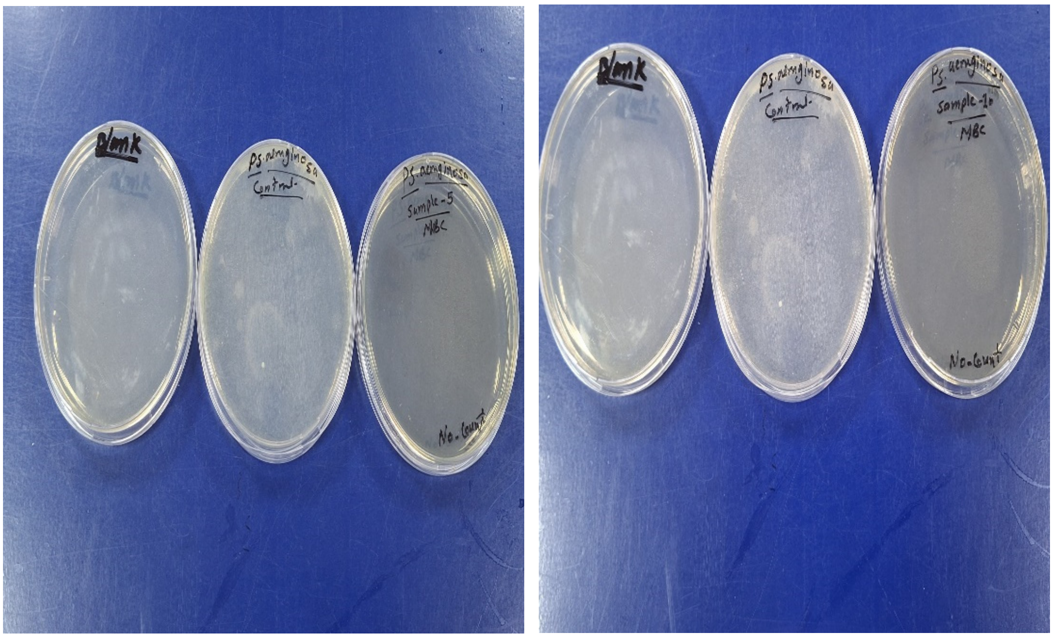

(A) Ethyl acetate fraction (B) Butanol fraction

Figure S10. Subculture photographs for Pseudomonas aeruginosa: (A) the ethyl acetate fraction and (B) the butanol fraction. Plates, left to right: blank, organism control, and treated sample. Original codes: 5, ethyl acetate fraction; 10, butanol fraction.

Additional views of antimicrobial endpoint plates

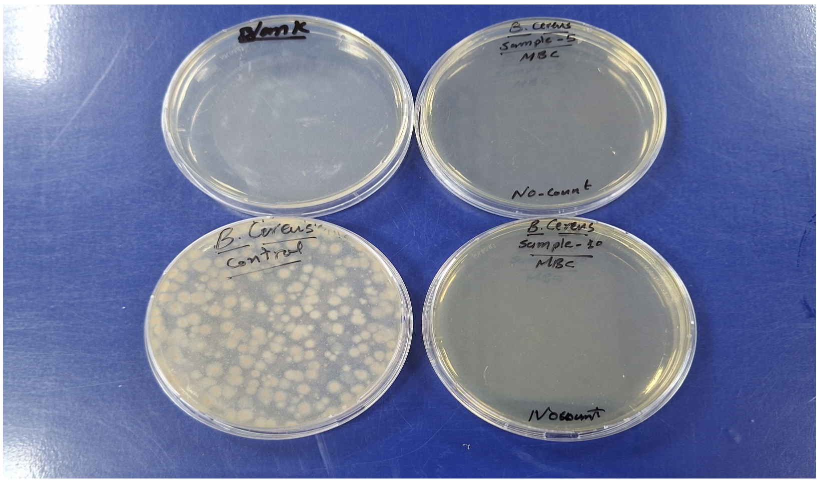

(A) Bacillus cereus

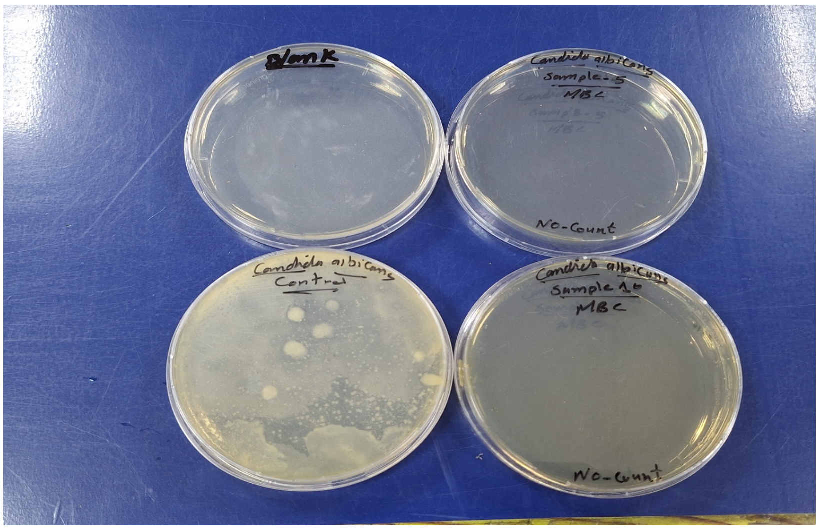

(B) Candida albicans

Figure S11. Additional photographs of subculture plates for (A) Bacillus cereus and (B) Candida albicans. Plate positions: upper left, blank; lower left, organism control; upper right, ethyl acetate fraction (5); lower right, butanol fraction (10).

Additional views of antimicrobial endpoint plates

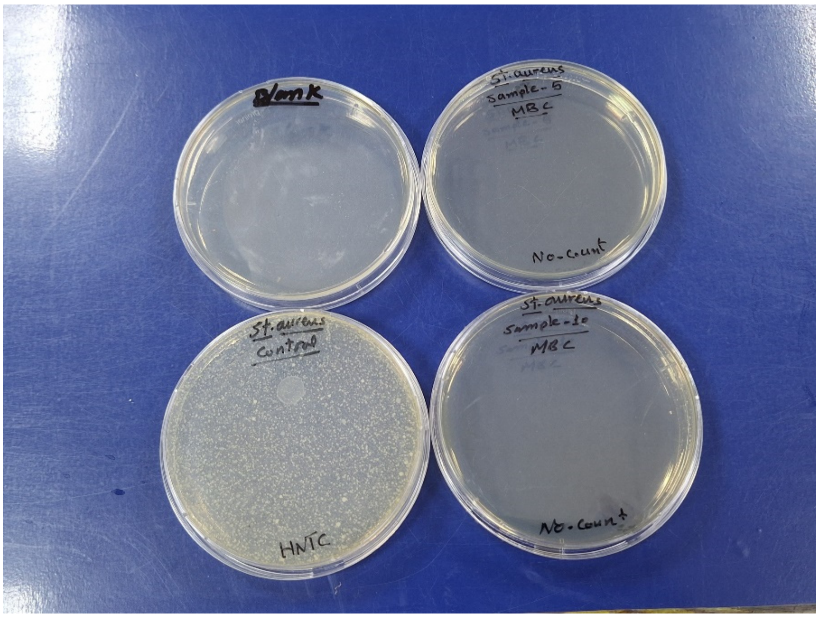

(A) Staphylococcus aureus

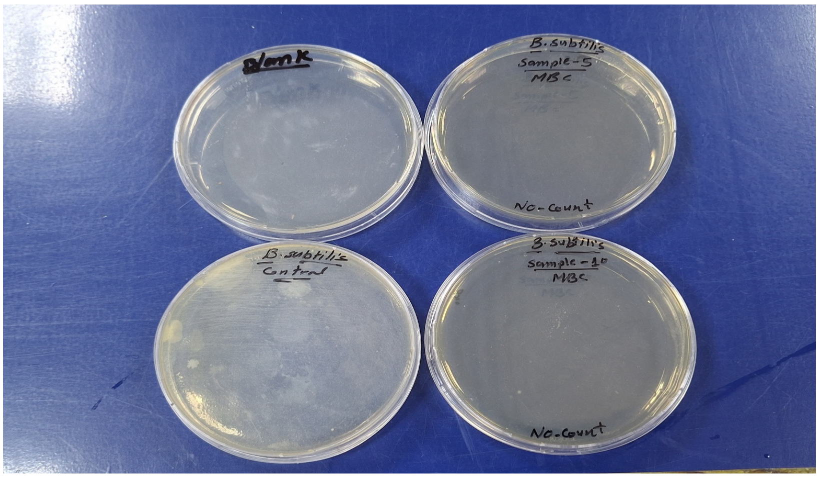

(B) Bacillus subtilis

Figure S12. Additional photographs of subculture plates for (A) Staphylococcus aureus and (B) Bacillus subtilis. Plate positions: upper left, blank; lower left, organism control; upper right, ethyl acetate fraction (5); lower right, butanol fraction (10).

Additional views of antimicrobial endpoint plates

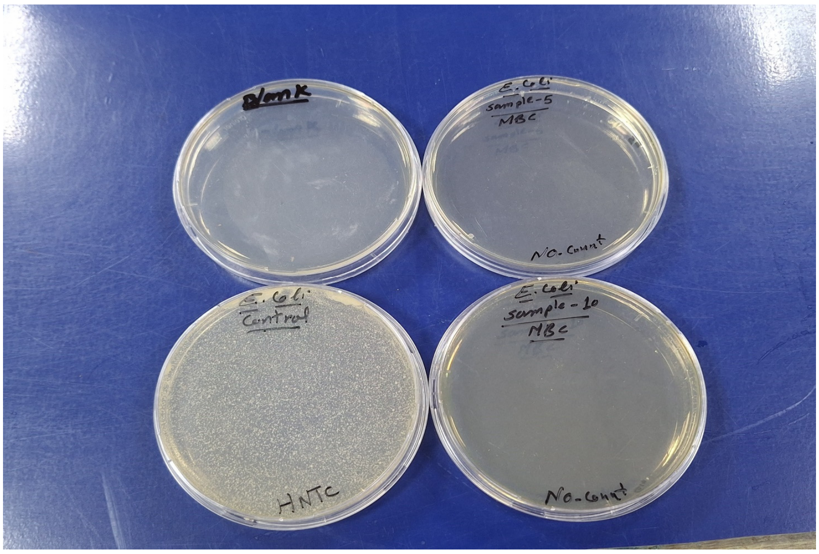

(A) Escherichia coli

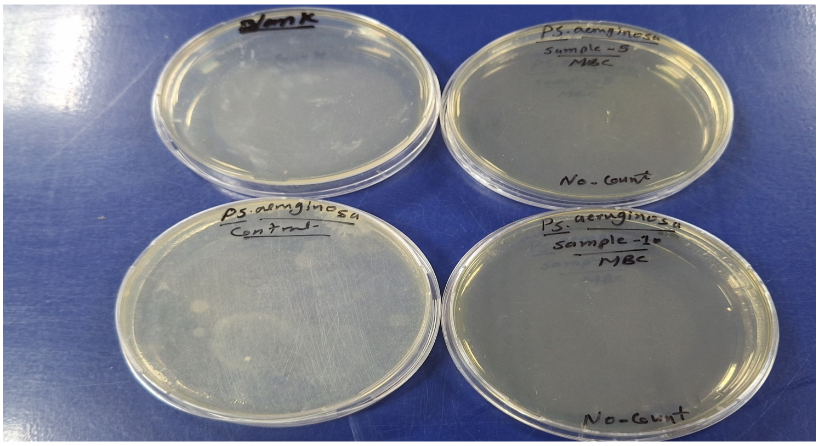

(B) Pseudomonas aeruginosa

Figure S13. Additional photographs of subculture plates for (A) Escherichia coli and (B) Pseudomonas aeruginosa. Plate positions: upper left, blank; lower left, organism control; upper right, ethyl acetate fraction (5); lower right, butanol fraction (10).
